# A widely distributed diheme enzyme from *Burkholderia* that displays an atypically stable *bis*-Fe(IV) state

**DOI:** 10.1038/s41467-019-09020-4

**Published:** 2019-03-07

**Authors:** Kimberly Rizzolo, Steven E. Cohen, Andrew C. Weitz, Madeline M. López Muñoz, Michael P. Hendrich, Catherine L. Drennan, Sean J. Elliott

**Affiliations:** 1 Boston University, Department of Chemistry, Boston, MA 02215 USA; 2 Massachusetts Institute of Technology, Department of Chemistry, Cambridge, MA 02139 USA; 3 Carnegie Mellon University, Department of Chemistry, Pittsburgh, PA 15213 USA; 4 Massachusetts Institute of Technology, Department of Biology, Cambridge, MA 02139 USA; 5 Howard Hughes Medical Institute, Cambridge, MA 02139 USA

## Abstract

Bacterial diheme peroxidases represent a diverse enzyme family with functions that range from hydrogen peroxide (H_2_O_2_) reduction to post-translational modifications. By implementing a sequence similarity network (SSN) of the bCCP_MauG superfamily, we present the discovery of a unique diheme peroxidase BthA conserved in all *Burkholderia*. Using a combination of magnetic resonance, near-IR and Mössbauer spectroscopies and electrochemical methods, we report that BthA is capable of generating a *bis-*Fe(IV) species previously thought to be a unique feature of the diheme enzyme MauG. However, BthA is not MauG-like in that it catalytically converts H_2_O_2_ to water, and a 1.54-Å resolution crystal structure reveals striking differences between BthA and other superfamily members, including the essential residues for both *bis-*Fe(IV) formation and H_2_O_2_ turnover. Taken together, we find that BthA represents a previously undiscovered class of diheme enzymes, one that stabilizes a *bis-*Fe(IV) state and catalyzes H_2_O_2_ turnover in a mechanistically distinct manner.

## Abstract (web summary)

The diheme enzyme MauG forms a *bis*-Fe(IV) state. Here the authors identify and determine the structure of BthA, a diheme peroxidase conserved in all *Burkholderia* and show that BthA also forms a *bis*-Fe(IV) species but mechanistically differs from MauG by combining magnetic resonance, near-IR and Mössbauer spectroscopies and electrochemical methods.

## Introduction

Microorganisms utilize a vast array of metal-containing proteins to protect the cell from reactive oxygen species (ROS). Proteins containing heme cofactors are well known for incorporating hydrogen peroxide (H_2_O_2_) in catalytic processes as a means to remove the xenotoxic oxidant generated via the incomplete reduction of dioxygen (O_2_)^1^. Within Gram-negative bacteria, cell viability is maintained in part by cytochrome *c* peroxidases (bCCPs), which convert H_2_O_2_ to H_2_O in the periplasm, taking electrons from small electron transfer proteins^2^. bCCPs are structurally and mechanistically distinct from well-studied monoheme plant and fungal peroxidases in that they house two *c-*type heme prosthetic groups covalently bound to the protein backbone by a CXXCH-binding motif^3,4^. However, the bCCP superfamily also includes MauG, an enzyme that shares the same canonical bCCP fold and many similarities in key amino acid positions, yet catalyzes strikingly different chemistry: where bCCPs only appear to be capable of oxidizing a redox partner protein of relatively low redox potential (~ 250 mV), MauG is responsible for the oxidation of specific Trp residues (~ 1 V) to form the catalytic cofactor tryptophan tryptophylquinone (TTQ) in the precursor protein of methylamine dehydrogenase (preMADH)^5–7^. A molecular perspective of how bCCPs and MauG differ in terms of the functionality as peroxidases has been long attributed to differences in the coordination environment of the coordinately saturated (6c) heme. Here, we further demonstrate that those differences are not the sole determiners of reactivity, and provide evidence of a class of diheme enzymes that share properties with classic bCCPs and MauGs, but is also distinct structurally and mechanistically.

**Fig. 1:**
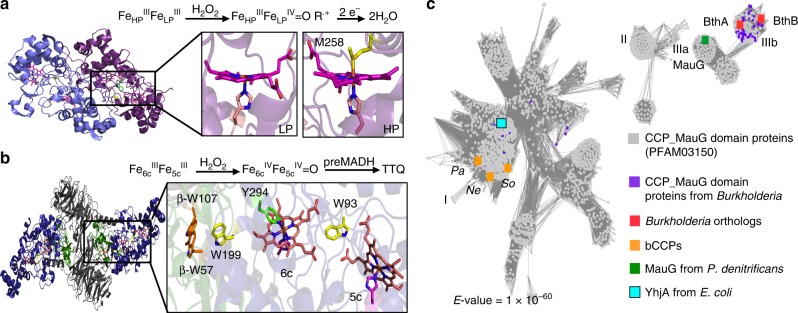
SSN of CCP_MauG domain containing proteins. **a** Active site domain of bCCPs. Reaction scheme indicates oxidation states achieved for reaction of bCCPs with H_2_O_2_. Upon addition of H_2_O_2_, a compound I intermediate is formed, where an electron is lost from a nearby tryptophan as is suggested for *Ne*. Crystal structure of *Ne* peroxidase (PDB: 1IQC) is shown as dimer (purple). Inset shows structure of the six-coordinate, high potential (HP) heme (right) and five-coordinate, low potential (LP) heme (left). **b** Active site domain of MauG. Reaction scheme shows overall oxidation state achieved for reaction of MauG with H_2_O_2_ in the presence of preMADH. Crystal structure of overall fold of MauG is shown in complex with preMADH (PDB: 3L4M). Inset shows diheme active site of MauG, including key tryptophan residues that are post-translationally modified in preMADH (β-W107, β-W57) after reaction with H_2_O_2_. Six-coordinate heme (6c) is shown with Tyr-His ligation. **c** SSN generated for CCP_MauG containing proteins with *E*-value = 1.0 × 10^–60^. Graphical representation of network created using Cytoscape. Main protein clusters, where each node represents and individual sequence, are identified by roman numerals. Cluster I includes canonical bCCPs, Cluster IIIa includes MauG from *P. denitrificans* and other known orthologs, and Cluster IIIb includes diheme enzymes (BthA and BthB from *B. thailandensis* are indicated) conserved in *Burkholderia*

In both MauG and bCCPs, the peroxidatic active site is a five-coordinate (5c), low potential (LP) heme, which serves as the site of H_2_O_2_ binding (Fig. 1a). However, the six-coordinate (6c), high potential (HP) heme site that assists in electron transfer (ET) to and from the active site differs with respect to the ligation environment. In bCCPs, such as the well-characterized enzyme from *Nitrosomonas europaea* (*Ne*)^8^, the (6c) heme is Met-His ligated, whereas in MauG, it is Tyr-His ligated^7^. These variations play out in terms of critical differences in chemistry, which correlates with the redox potentials observed when reducing equivalents are introduced into the protein system. In canonical bCCPs, the presence of the Met-His ligated HP heme means that the enzyme can be prepared in a very stable Fe_HP_^II^ Fe_LP_^III^ oxidation state, which contrasts with MauG. Instead, the two hemes of MauG are close in redox potential^6^ and are termed 5c and 6c accordingly; the Fe_5c_^II^Fe_6c_^III^ form of the enzyme is not stable, which appears to be critical for MauG function.

MauG’s function is the six-electron oxidation of two tryptophan residues (βTrp57 and βTrp107) in preMADH, achieved by long-range ET through the two heme sites of MauG, a precise and long-distance chemistry that earned MauG the moniker ‘Nature’s sniper’ (Fig. 1b)^9–11^. Studies of MauG have focused on the characterization of a *bis-*Fe(IV) species, formulated as Fe^IV^Fe^IV^ = O, which is formed upon reaction with H_2_O_2_ in the absence of substrate, preMADH^12^. The *bis-*Fe(IV) state is a unique alternative to the extensively studied compound I (Cpd I) of monoheme cytochrome P450 enzymes and monoheme plant peroxidases^13,14^. Currently, the proposed mechanism of MauG involves H_2_O_2_ binding to the 5c heme to generate an unstable Cpd I species, which is immediately reduced to Cpd II via oxidation of the 6c heme to Fe(IV), while still maintaining Tyr-His ligation^12^. The formation of the *bis-*Fe(IV) state has set MauG apart from the canonical bCCPs and, more excitingly, highlights the diverse chemistry present in microorganisms.

We have taken a bioinformatics approach to aid a comparison of MauG structure and function with respect to canonical bCCPs, and in doing so, we have identified family members of unusual reactivity. MauG is encoded by one of 11 genes present in the *mau* cluster^15^, and in looking for close relatives of MauG, (Fig. 1c), we recognized an uncharted region of potential H_2_O_2_ reactivity, obligately found in soil microbes of the genus *Burkholderia*, an opportunistic bacterium, with known implications in serious diseases such as cystic fibrosis^16^. Herein we report the characterization of the class A diheme enzyme BthA (Uniprot ID: Q2T6B0) from *B. thailandensis*. Our efforts reveal that BthA is a peroxidase, capable of both H_2_O_2_ turnover when in the presence of an external electron source, yet also generates the *bis*-Fe(IV) species, as determined by Near-infrared  (NIR) and Mössbauer spectroscopy. Despite the similarities between BthA and MauG reactivity shown below, we find that the genomic context of the *bthA* gene reveals it is not encoded within a *mau* operon. While *bthA* is highly conserved in all strains of *Burkholderia* it is always found downstream of a putative phosphatase partner of unknown function (Supplementary Fig. 1), but with annotation that indicates extra-cellular localization. *bthA* is predicted to be localized in the periplasm, as it contains a canonical leader sequence associated with the periplasmic maturation of *c*-type cytochromes in Gram-negative microorganisms. Intriguingly, a 1.54-Å resolution crystal structure presented herein confirmed Tyr-His ligation of heme 6c, yet surprisingly, revealed lack of a clear ET network of residues between the two heme sites, assumed to be essential for generation of a *bis-*Fe(IV) state. Unlike the canonical tryptophan residue present midway between the heme centers of both bCCPs and MauG, the BthA structure provides evidence for a serine residue in this highly conserved position, which was shown not to inhibit the *bis-*Fe(IV) formation or peroxidase activity when mutated to alanine. Taking a biochemical and biophysical approach to exploring uncharacterized members of the peroxidase superfamily, we introduce a diheme enzyme to the peroxidase superfamily involved in mechanistically distinct oxidation chemistry.

## Results

### A sequence similarity network of the CCP_MauG superfamily

A SSN of the bacterial peroxidase superfamily was generated by use of the enzyme function initiative (EFI) sequence similarity tool^17,18^. Our network comprises protein sequences found with PFAM domain CCP_MauG, identified with the code PF03150. Pooling over 6000 protein sequences, *E*-values were lowered in steps of 10^–10^ to arrive at a stringent *E*-value cutoff value of 10^–60^, which results in a representative isofunctional network possessing several protein-sequence clusters, where each node represents a set of proteins with 80% or higher sequence identity (Fig. 1c). Using Cytoscape^19^ to represent the network graphically, canonical bCCPs were mapped to Cluster I, which includes the structurally characterized enzymes from *Ne, Pseudomonas aeruginosa* (*Pa*), and *Shewanella oneidensis* (*So*), despite known differences in redox-based activation for these bCCP members^8,20,21^. The main cluster also contains triheme peroxidase YhjA from *E. coli*, which has recently been reported to use H_2_O_2_ as a respiratory substrate under anoxic conditions^22^. The map of proteins conserved in the different clusters of the network suggests a difference in protein function as the primary sequence of the peroxidases evolve. As suspected, MauG from *P. denitrificans* is located in a separate cluster from the canonical bCCPs (Cluster IIIa, Fig. 1c). True MauG orthologs, where genome analysis provides evidence for a *mau* operon, are largely found in Cluster IIIa. Surprisingly, these MauG orthologs are also connected to Cluster IIIb, which represents sequences that share ~ 30% identity to MauG and ~25% identity to the canonical bCCPs of Cluster I (See Supplementary Table 1). These sequences contain two CXXCH motifs, are found obligately in the genus *Burkholderia*, and further analysis of Cluster IIIb identified two separate diheme proteins present in all *Burkholderia* strains. Exploration of the *Burkholderia* genomes revealed lack of a conserved *mau* operon, suggesting an alternate chemical role for these diheme bCCPs. We term these two related *Burkholderia* proteins as Class A (found on chromosome II), and Class B (chromosome I). A sequence alignment of the A and B enzyme from *B. thailandensis* shows a 40% sequence identity between the proteins. Both BthA and BthB possess the conserved tyrosine residue predicted to ligate the 6c heme site, and yet have clear differences with MauG or other bCCP family members: their N-termini are rich in alanine residues, suggesting hydrophobicity (though signal prediction servers suggest both are exported periplasmically), and both enzymes possess conserved cysteine residues near their C-termini (and the structure presented below reveals an unforeseen disulfide linkage). These traits may yield clues to better understand the H_2_O_2_ chemistry observed for BthA.

### BthA as a redox mimic of MauG

**Fig. 2:**
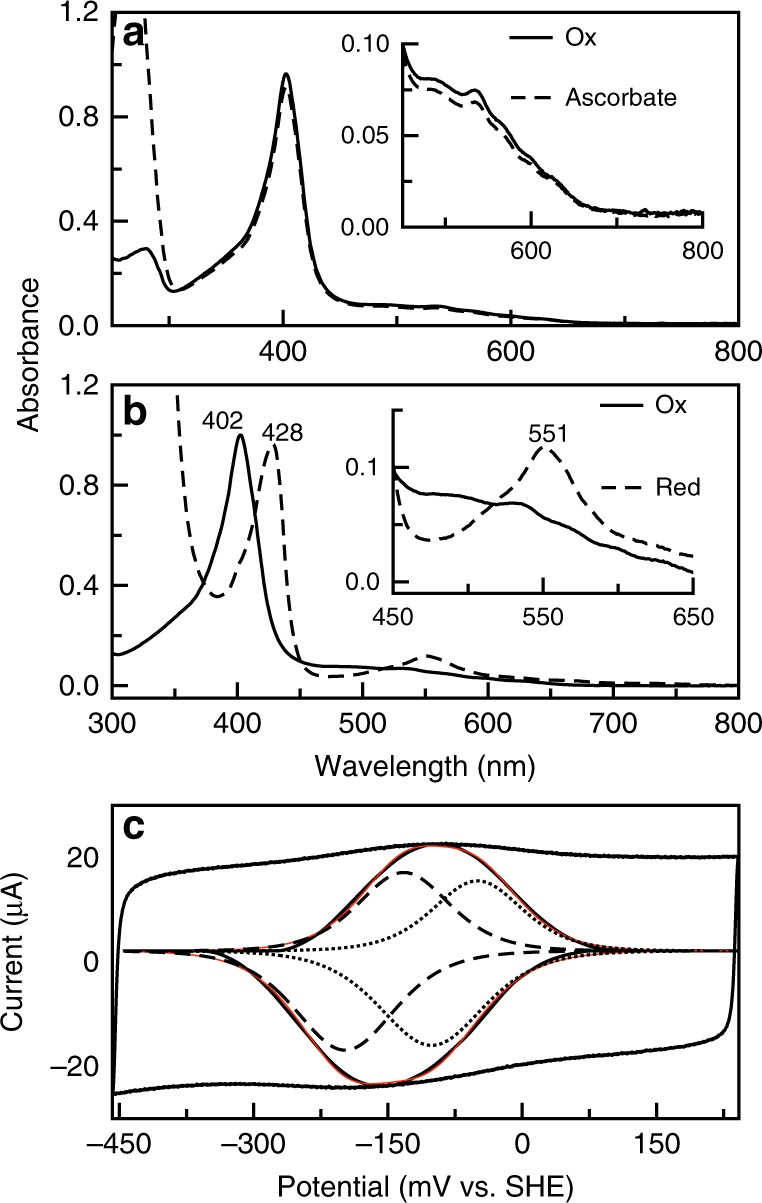
Optical characterization of BthA. **a** Oxidized BthA (solid line) and ascorbate treated (dashed line). Ascorbate was added to 20 mM final concentration before scan was collected. Inset shows range from 450 to 800 nm, indicating no signs of reduction to ferrous form. **b** Oxidized BthA (solid line) and dithionite treated (dotted line). Dithionite was added to a final concentration of 1 mM. Inset shows peak formed at 550 nm. **c** Deconvoluted spectra of BthA on PGE. Voltammogram of BthA on MWCNT-PGE at pH 7, 21 °C, 100 mV s^−1^. Raw voltammogram is shown (black) with the baseline subtracted (black, inset) with fit for the overall signal (red) and individual species (dotted and dashed line)

Establishing the optical and electrochemical properties of BthA is important for establishing how this diheme enzyme relates to known bacterial peroxidases. BthA purifies with both heme irons in the oxidized state (diferric), with a Soret maximum at 403 nm, and a broad feature centered at 630 nm believed to signify a high-spin, five-coordinate heme site. Additionally, BthA has four distinct peaks at 495, 538, 570, and 610 nm, which we attribute to Tyr-His ligation for six-coordinate ferric heme centers based on similar literature values reported for the heme-binding protein HasA^23^. A reductive titration with up to 20 mM ascorbate added to the diferric state results in no spectral changes, highlighting an inability of BthA to achieve the optically characterized semi-reduced (Fe_HP_^II^ Fe_LP_^III^) state observed for canonical bCCPs achieved when treated with only 1 mM sodium ascorbate (Fig. 2a). Upon reduction with dithionite, the electronic absorption spectrum of BthA is atypical of canonical bCCPs and MauG, particularly with respect to the α/β band region (Fig. 2b). Reduction of BthA results in a Soret shift from 403 to 428 nm, and generates a broad feature at 550 nm, as opposed to sharp α/β bands at 550 and 524 nm, respectively, reported for most reduced cytochrome *c* proteins^24^. Dithionite-reduced BthA was shown to bind the exogenous ligand carbon monoxide (CO), demonstrated by (1) a blue shift in the Soret to 416 nm; (2) increase in extinction coefficient; and (3) formation of a broadened band in the α/β band region at 550 nm. Collectively, these data suggest a closer relationship of BthA to MauG than any known bCCP; a salient finding here is the inability of ascorbate to reductively generate a Fe_HP_^II^ Fe_LP_^III^ state with a diagnostic Soret shoulder at 414 nm and the partial appearance of α/β bands^8^.

The electrochemical properties of the heme centers of BthA were determined in order to correlate the reduction potentials to the spectroscopic features observed for diferric and diferrous forms of the protein. Both direct electrochemistry and coupled optical titrations revealed two redox couples that are close in potential, such that producing a single semi-reduced state (i.e., like bCCP) is unlikely if not impossible. By use of protein film voltammetry (Fig. 2c), the redox characteristics of the two heme centers can be fit to a model of two species with potentials of −121 and −165 mV vs. SHE, consistent with (though not identical to) what has been observed for MauG, when the protein is reduced with transitions occurring at −159 and −244 mV vs. SHE^6^. Parallel spectroelectrochemical titrations utilizing an optically transparent thin-layer electrode (OTTLE)^25^ yielded similar BthA redox transitions of −100 and −220 mV (Supplementary Fig. 2).

### Verification of MauG-like heme environments in the resting state

**Fig. 3:**
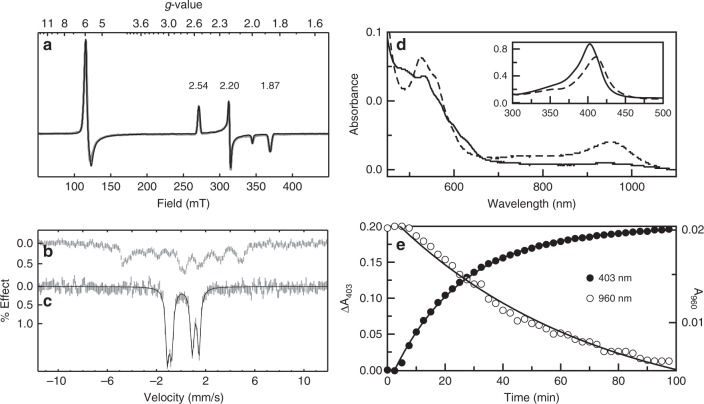
Formation of a *bis-*Fe(IV) species in BthA. **a** EPR spectrum (9.642 GHz, 2 μW) of 0.8 mM as-isolated Wt BthA recorded at 13 K. See main text for simulation parameters and corresponding species concentrations. **b** Mössbauer spectrum and simulations of as-isolated ^57^Fe-enriched BthA recorded in an applied field of 45 mT parallel to the incident γ-radiation, at a temperature of 4.2 K. See main text for simulation parameters. **c** After addition of 10 equiv. H_2_O_2_ to the sample in **b**. **d** NIR spectra of Oxidized BthA (solid line) and H_2_O_2_ treated (dashed line). After addition of H_2_O_2_, scans were collected every 2 min for 1.5 h (gray scans). The inset shows the shift in Soret from 403 to 411 nm. Over time, the feature at 960 nm begins to decay, and the Soret shifts back to the 402 nm position, suggesting reduction back to the initial, diferric state. **e** Decay of the *bis-*Fe(IV) monitored by the change in absorbance for the 402 and 960 nm feature vs. time. The rise of the Soret back to the 403 nm (closed circles) and the 960 nm decay (open circles) are shown as a function of time. Data were fit to a single exponential rise/decay

As indicated by the absorption spectrum for as-isolated protein, BthA purifies in the diferric state and both heme centers were found to be active by electron paramagnetic resonance (EPR) spectroscopy (Fig. 3a). The X-band EPR spectrum of BthA is similar to MauG, displaying two distinct heme centers as a mixture of high- and low-spin species. Quantitative simulations of the EPR spectra are overlaid on the experimental spectrum (Fig. 3a). The high-spin (HS) species gave *g-*values near *g* = 6 and 2, indicative of an axial (*E*/*D* < 0.001) 5-coordinate (5c) heme, accounting for 45% of the Fe(III) in the sample. The low-spin (LS) species had *g*-values of 2.54, 2.19, 1.86, accounting for 55% of the Fe(III) in the sample. The total heme concentration was in agreement with the protein concentration. The observed ratio of the two species can be dependent on the method of purification, wherein some of the 5c site heme converts to 6c as a function of pH.

The Mössbauer spectrum of diferric BthA in a field of 45 mT recorded at 4 K is shown in Fig. 3b. The spectrum showed a mixture of *S* = 5/2 and *S* = 1/2 Fe(III) species in approximately the same ratio as observed from the EPR spectrum. However, without accompanying high-field data, a unique fit for the HS and LS Fe(III) hemes could not be obtained^26^. At higher temperature (100 K), the electronic relaxation was intermediate relative to the nuclear precession frequency and gave a broad featureless spectrum.

### BthA produces a *bis*-Fe(IV) species like MauG

To date, MauG is the only reported diheme enzyme capable of forming a *bis-*Fe(IV) state when reacted with H_2_O_2_^12^. Optically, generation of this state can be monitored via formation of a peak at 950 nm, defined by a charge resonance stabilization between the 5c and 6c hemes due to the reversible oxidation and reduction of Trp93 located midway between the hemes^27^. This  NIR feature occurs concomitant with a shift in Soret and growth of charge transfer (CT) bands. The lifetime of the *bis-*Fe(IV) species in MauG was 10 min, and the reduction back to the diferric state was achieved by oxidative damage of nearby Met residues associated with the 5c heme^28^. BthA is also capable of generating the putative *bis-*Fe(IV) species and produces an electronic absorption spectrum similar to peroxide-treated MauG (Fig. 3d). Upon treatment of BthA with H_2_O_2_ a peak at 960 nm immediately appeared coupled with a decrease in Soret intensity and shift from 403 to 411 nm in addition to the appearance of CT bands at 524 and 560 nm. However, we found two striking differences between this newly generated species of BthA when compared to MauG. First, the rate for enzyme relaxation back to the diferric state (as monitored by the Soret position and intensity) is markedly slower than MauG. Monitoring the decay of the 960 nm feature and return of Soret to 403 nm, this oxidized state can persist for over one hour (Fig. 3e). The decay of the 960 nm peak was fit to a single exponential rate constant of 0.014 min^−1^, whereas the 403 nm peak was fit to a single exponential rise with a rate constant of 0.04 min^−1^. As discussed below, this observation challenges the convention of the proposed reactivity of a Fe^IV^Fe^IV^ = O species as a hot oxidant.

The long-lived nature of the NIR feature at 960 nm prompted further investigation of this unusually stable *bis*-Fe(IV) state by EPR and Mössbauer spectroscopies. Addition of one equivalent H_2_O_2_ to an EPR sample of BthA resulted in a 50% loss of signal intensity for both the HS and LS features, suggestive of an oxidative event of each heme site to an EPR-silent Fe(IV) state. Samples of ^57^Fe-enriched BthA were treated with H_2_O_2_ and Mössbauer spectra were recorded at 4 K in a field of 45 mT. Upon exposure to one equivalent of H_2_O_2_, 50% of the diferric signal was lost and replaced by two doublets having approximately equal areas and parameters: (1) *δ* = 0.07 mM s^−1^, ΔEq = 1.70 mM s^−1^; and (2) *δ* = 0.17 mM s^−1^, ΔEq = 2.58 mM s^−1^. Addition of excess H_2_O_2_ (10 equiv.) resulted in complete conversion of the HS and LS species to the two new doublets (Fig. 3c). The parameters were indicative of the formation of Fe(IV) species and were similar to those of MauG^12^. Thus, in terms of optical and magnetic spectroscopies, a key similarity between BthA and MauG was established based on the presence of a *bis*-Fe(IV) species, which possesses kinetic stability over the course of minutes.

### BthA as a potential peroxidase

Given the remarkable electronic similarity between BthA and MauG, the absence of a *mau* operon to support BthA’s function and ubiquity in *Burkholderia* prompted us to investigate its potential enzymatic activity. MauG is considered a poor peroxidase^5^, and BthA shares only a ~ 25% sequence identity with other canonical bCCP family members (e.g. enzymes from *Ne* or *Geobacter sulfurreducens*)^29^. Catalytic, steady-state reduction of H_2_O_2_ was assayed to address the putative role of BthA in H_2_O_2_ detoxification. Surprisingly, the kinetic parameters of H_2_O_2_ turnover indicate BthA is a very good peroxidase despite its spectroscopic and electrochemical parallels to MauG. For comparison to activity reported for bCCPs, activity was first determined with use of commercially available cytochrome *c* from horse heart (HH cyt *c*) (reduced) as the electron donor, but no trace of activity was detected. It is possible the molecular interaction between BthA and cyt *c* is not favorable and, therefore, resulted in poor electron transfer.

Using ABTS as the artificial electron donor, activity was measurable. For BthA, *k*_cat_/*K*_M_ = 1.6 × 10^6^ M^−1 ^s^−1^ with a relatively low *K*_M_ of 3.4 ± 0.4 µM. These values are comparable to canonical bCCPs with *k*_cat_/*K*_M_ values that range from 10^6^–10^8^ M^−1 ^s^−1^. To validate this assay, we mutated a conserved residue near the 5c heme (Glu277 in BthA) that is known to act as an acid-base catalyst during peroxide turnover^30^. We found that the E277Q variant was inactive compared to the Wt enzyme (Supplementary Table 2), providing further support it is peroxidase activity that is being reported by ABTS. Additionally, the E277Q enzyme variant was unable to generate the *bis-*Fe(IV) species when analyzed by NIR spectroscopy (Supplementary Fig. 3), a finding also reported for the E113Q variant of MauG^31^.

### Structure of BthA reveals key differences from both bCCPs and MauG

**Fig. 4:**
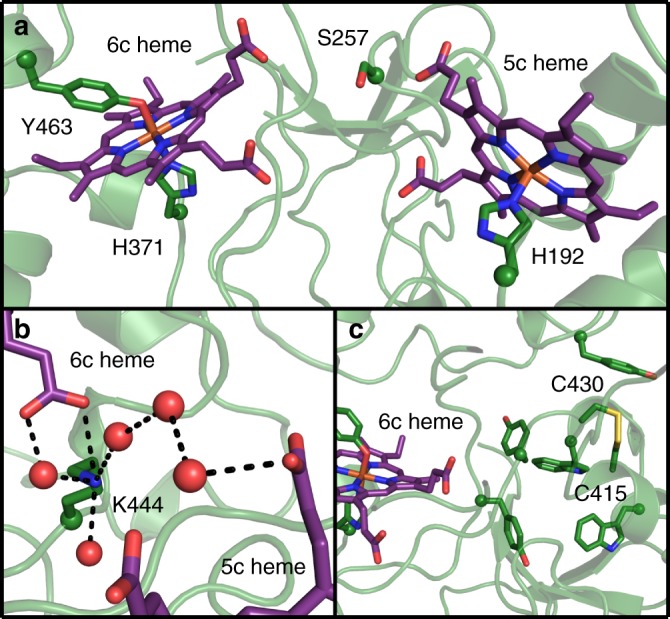
Structure of BthA. **a** Ser257 is positioned between the 6c heme, coordinated by His 371 and Tyr463, and the 5c peroxidatic heme, coordinated by His 192. **b** Lys444 mediates a water network between the propionate groups of the 5c and 6c hemes. **c** An intramolecular disulfide between Cys415 and Cys430 is located near the 6c heme and is surrounded by tyrosine and tryptophan residues. Peptide and heme carbon shown in green and purple, respectively. Oxygen, nitrogen, sulfur, and iron shown in red, blue, yellow, and orange, respectively. Peptide backbone shown in cartoon representation and sidechains and hemes shown in stick representation. Side-chain α-carbon and water shown as spheres

The structure of BthA was solved using X-ray crystallography to 1.54-Å resolution (Supplementary Table 3), revealing the same overall fold found in other structurally characterized bCCPs and MauG (Supplementary Fig. 4). The Cα rmsd of BthA with MauG (PDB 3L4M, 34% sequence identity)^7^ and *Ne* bCCP (PDB 1IQC, 25% sequence identity)^4^ are 0.974 Å and 3.775 Å, respectively. The structural data further show that the 5c heme pocket is conserved with both bCCPs and MauG, including the presence of the acid-base catalyst, Glu277, which was mentioned above (Supplementary Fig. 5). The 6c heme pocket in BthA is more similar to MauG, showing Tyr-His ligation by Tyr463 and His 371 (Fig. 4a and Supplementary Fig. 5). Mutation of Tyr463 to histidine (Y463H) results in the inability of the enzyme to generate the NIR feature when treated with H_2_O_2_ (Supplementary Fig. 3), a finding reported for the Y294H mutation of MauG^32^. Yet, Y463H maintained H_2_O_2_ reduction when in the presence of an external electron donor with kinetic values comparable to Wt (Supplementary Table 2**)**.

Both MauG and previously characterized bCCPs contain a tryptophan residue positioned between both hemes^7,20,29,33–35^. Structural and kinetic studies of MauG have shown this interheme tryptophan residue to be necessary for *bis-*Fe(IV) stabilization and TTQ biosynthesis. In place of this interheme tryptophan residue, BthA has a serine residue, Ser257 (Fig. 4a and Supplementary Fig. 6). We addressed whether this serine residue plays a role in *bis-*Fe(IV) species formation through mutational analysis, creating S257A to eliminate any possible involvement in ET, and S257W to mimic the conserved tryptophan. Importantly, both S257A and S257W variants generated the *bis-*Fe(IV) species based on detection of the 960 nm feature in NIR and yielded nearly identical kinetic values for peroxide turnover (Supplementary Table 2 and Supplementary Fig. 3). A final trait common to MauG and bCCPs is a catalytically essential Ca^2+^ ion located between the propionate moieties of the two hemes. This ligand maintains an ordered solvent network between the two heme cofactors, and is proposed to regulate the reduction potential and coordination chemistry of the two hemes during catalysis^36–38^. The Ca^2+^ ion in MauG is tightly bound through coordination to four water molecules, the main-chain carbonyl oxygen atoms of Thr275 and Pro277, and the oxygen atom of the Asn66 side-chain. Additionally, the propionate A carboxylate oxygen of heme 6c is hydrogen bonded to two of the waters that provide Ca^2+^ ligands. The arrangement of these key residues and heme propionate group is identical for both bCCPs and MauG. In place of a calcium ligand, BthA contains a lysine residue, Lys444, which mediates a similar solvent network between the heme cofactors (Fig. 4b and Supplementary Fig. 7). Notably, the crystal structure revealed the presence of a unique surface-exposed disulfide bond between residues Cys415 and Cys430 (Fig. 4c). Sequence alignment of the *Burkholderia* orthologs shows the presence of these two cysteine residues at the C-terminal end of the protein sequence is conserved across all Class A proteins. Currently, the function of this disulfide is unknown, but it also sets BthA apart from canonical bCCPs.

## Discussion

Generation of a SSN for the bacterial peroxidase superfamily sets the foundation for discovery of complex peroxide induced chemistry of heme containing proteins. The network shows conservation of canonical bCCPs within the main cluster, representing bCCPs that require reductive activation as well as those reported as constitutively active. The nearest protein neighbors to the biochemically characterized MauG enzyme from *P. denitrificans* appear to be highly conserved in Gram-negative soil bacteria with genomic context suggesting an entirely different area of peroxidatic chemistry. The genomic context of the uncharacterized proteins in the network suggest changes in protein function that extend beyond the traditional role in H_2_O_2_ detoxification, or in the case of MauG, TTQ formation.

BthA is the first example of a diheme enzyme carrying out chemistry reminiscent of both canonical bCCPs and MauG. The electrochemical similarities between BthA and MauG suggest similar propensity for redox chemistry: the voltammetric data collected by direct electrochemistry revealed that both hemes must be similar in potential (where formal potentials arrive from fits to two one-electron redox couples of values of −65 and −121 mV), like the two redox transitions observed for MauG. These redox traits may be required to allow for the two electron, metal-centered chemistry associated with the production of the *bis*-Fe(IV) intermediate. However, the BthA redox potentials are higher than the transitions reported by MauG by 30–55 mV. The small difference between the individual 5c and 6c heme reduction potentials appears to correlate with the observed generation of the *bis-*Fe(IV) species. However, the slower decay rate of that spectroscopically observed species in BthA may be linked to the difference in the BthA redox potentials in comparison with MauG.

Crystallographic data on BthA shows MauG-like Tyr-His ligation of heme 6c and a similar environment around heme 5c as has been observed in both bCCPs and MauG. Mutational analysis of both the 6c heme site and the 5c heme site provided insight into the dual peroxide reactivity of BthA. Y463H and Y463M variants were incapable of generating a feature at 960 nm when reacted with H_2_O_2_, but surprisingly peroxidase turnover was unchanged in the ABTS-linked assay (Supplementary Table 2). The presence of a Tyr-His ligation to 6c heme site in BthA appears essential for directing BthA toward *bis-*Fe(IV) formation, but not does not appear to impact the ET mechanism required for H_2_O_2_ reduction (i.e., peroxidase activity with an artificial electron donor). However, E277Q, at 5c heme site, impairs both the *bis-*Fe(IV) formation and peroxidase activity. The position of this glutamate residue (Glu277) in the distal pocket of the 5c active site of BthA is highly conserved alongside a glutamine residue (Gln267) and proline residue (Pro271), which are analogous to Gln103 and Pro107 in MauG and have been shown to greatly impact heme conformation and reactivity of MauG^39,40^. Conservation of these three distal pocket residues across bCCPs, MauG and BthA indicates variation of this site is detrimental to the initial peroxide-binding step of either *bis-*Fe(IV) formation or H_2_O_2_ reduction.

Despite the similarity in protein fold, when comparing BthA and MauG, the lack of a tryptophan residue midway between heme 5c and 6c in BthA is a remarkable finding as this feature has been suggested to be critical for both *bis*-Fe(IV) formation in MauG, and the peroxidase activity of bCCPs^30^. Ser257 replaces the highly conserved Trp93 of MauG, and the replacement of an aromatic side-chain with an aliphatic one would suggest impeded electron transfer. Using the computational tool of HARLEM (see *Methods*), we examined the potential ET pathways between the 6c and 5c hemes, considering the electron transfer as either a set of two steps that relay through the position equivalent to Ser257 (Trp93 in MauG) or as a single longer step from 6c heme to 5c heme, as has been conducted previously for MauG^27^. Through this approach it is clear that replacement of tryptophan with serine impedes ET, as a direct path for a redox relay to occur is compromised. When considering ET as a series of two steps, the calculated electronic coupling between the 6c heme and Ser257 is approximately tenfold lower than the analogous step from heme 6c to Trp93 in MauG (*H*_AB_ is 3.5 × 10^–3^ for BthA, and 3.7 × 10^–2^ for MauG). In this model, the second step also has a reduced *H*_AB_ term, though only by a factor of two (1.4 × 10^–2^ for BthA, compared to 3.8 × 10^–2^ in MauG). As *k*_et_ is proportional to (*H*_AB_)^2^, the loss of electronic coupling by a factor of 10 in the first step should greatly impact overall ET rates.

And, when considering the single-step process, HARLEM predicts that direct ET from 6c to 5c hemes in BthA occurs through a circuitous route (Supplementary Fig. 8), where the heme proprionate of the 6c heme serves as pathway for communication to the 5c heme via the backbone residues of His449 and Arg244. This predicted single-step process aligns well with our results for the Ser257 variants, where we still observe a NIR feature upon addition of H_2_O_2_,(Supplementary Fig. 3) and similar steady-state kinetic rates compared to Wt (Supplementary Table 2). While significant work remains to assess the differences in ET traits between BthA and MauG and how electronic communication may be necessary for *bis-*Fe(IV) formation, we note that ET between the hemes, therefore, must occur. We then conclude BthA likely reduces H_2_O_2_ with a mechanism distinct from canonical bCCPs.

The lack of a Ca^2+^-binding site in the structure of BthA is also notable in that Ca^2+^ is known to be essential for enzymatic activity of bCCPs and MauG^37^. In MauG, the presence of Ca^2+^ has been shown to impact the heme conformation and spin state, which ultimately impairs the ET that occurs between the heme sites^41^. However in the BthA structure, the ε-ammonium group of Lys444 occupies the position of the conserved Ca^2+^ ion, one of the notable differences between BthA and MauG, suggesting that the positively charged Lys side-chain mimics a Ca^2+^ ion. We conducted mutational analysis of Lys444, surveying the affect of a buried charged residue on protein stability and H_2_O_2_ reactivity, and confirming our hypothesis that the positive charge supplied from the ε-ammonium group of Lys444 is essential, effectively replacing the role of Ca^2+^ observed in other bCCP family members. We produced a K444R variant, which maintains a positive charge at the same position, and showed this variant behaved like Wt and was capable of both H_2_O_2_ reduction (specific activity of 5.3 µmol H_2_O_2 _min^−1^ mg^−1^ compared to Wt with 6.1 µmol H_2_O_2 _min^−1 ^mg^−1^) and the production of the NIR feature synonymous with the *bis-*Fe(IV) state (Supplementary Fig. 3). However, alteration of the positive charge at this position was deleterious: mutating Lys444 to glutamine (charge removal) or glutamate (charge reversal) resulted in insoluble protein in both cases, suggesting Lys444 is critical for stability if not chemistry.

The Ca^2+^ ion in MauG is tightly bound through coordination to the main-chain carbonyl oxygen atoms of Thr275 and Pro277, the oxygen atom of the Asn66 side-chain, and four additional water molecules, an arrangement also highly conserved in bCCPs. For BthA, the presence of the Lys444 is in the position of the conserved threonine residue and Ser446 is in place of the highly conserved proline residue. Both seem equally effective in generating a network of water molecules between the two hemes, but any regulation that is based on Ca^2+^ ion concentrations would be lost in BthA. However, two C-terminal cysteines, which form a disulfide in the BthA structure and are widely conserved in the primary sequence of proteins present in the *Burkholderia* cluster of the SSN, present a feature of the bCCP family of possible regulatory significance. Disulfide formation can be structural, regulatory or catalytic, and is not a common feature of the bacterial peroxidase family, and thus represents an interesting opportunity for further study.

While the function of BthA has not yet been discovered, the conservation of BthA and the traits of its expression provide hints as to potential biochemical roles. As *Burkholderia* strains lack conserved *mau* operons, it is clear that BthA cannot act as a true MauG. It is always found in proximity to a conserved putative purple-acid phosphatase (*phosA)*, which is predicted to be localized to the outward face of the outer-membrane, and it is conceivable that BthA carries out potential oxidation chemistry on this putative protein partner. Transcriptional analysis by quantitative PCR (qPCR) showed an overexpression of both *bthA* and *phosA* ( + 5 and + 14, respectively) during anaerobic growth of *B. thailandensis* as compared to aerobic growth. This preliminary result helps support our hypothesis that the function of BthA extends beyond a traditional role in H_2_O_2_ scavenging, where H_2_O_2_ is typically produced internally as a misfire of respiration under aerobic conditions.

Generation of a bacterial peroxidase SSN allowed us to biochemically map where canonical bCCPs and MauG lie within the peroxidase superfamily, and identify an area of uncharacterized peroxide chemistry present in *Burkholderia*. By use of bioinformatics and biophysical methods, we have identified a previously unreported diheme enzyme that stands out from the traditional bCCPs and MauG enzymes. Probing the ET network of BthA, we have identified some of the residues essential for the directing the enzyme toward *bis-*Fe(IV) formation, while maintaining the ability to efficiently reduce H_2_O_2_.

## Methods

### Sequence similarity network for CCP_MauG domain proteins

A sequence similarity network was generated using the sequence similarity tool available through EFI. The sequences input for computation are represented with Pfam identifier for CCP_MauG family members, code PF03150. The network was generated using the InterPro 58 and UniProt 2016_06 databases, and consisted of 6600 sequences with the PF03150 identifier. Final network displayed is representative of an alignment score of 30 and  *E*-value cutoff of 10^–60^, with  each node representing sequences with 80% sequence identity. Cytoscape^19^ was used for the graphical representation of the network.

### Molecular biology

*bthA* from *Burkholderia thailandensis* (gene ID: BTH_II1092) was synthesized and codon optimized (Supplementary Table 4) for expression in *E. coli* (GeneWiz). BTH_II1092 was sub-cloned into pETSN expression vector with a cleavable OmpA periplasmic leader sequence and T7 promoter^29^. Cloning was performed routinely using restriction enzymes *Xho*I and *Eco*RI, purchased from New England Biolabs (NEB), plasmid mini-prep kits (Qiagen) and T4 DNA ligase (NEB). To avoid potential complications from export to the periplasm and maturation of cytochromes *c*, a truncated BthA sequence, starting at S69 on the N-terminal end was generated. The plasmid (pKR2) contains an N-terminal Strep tag and C-terminal 6xHis tag. The pKR2 vector was further modified to remove the N-terminal Strep tag, and to insert a stop codon and delete the 6xHis tag, generating pKR4, an untagged version of BthA (Supplementary Table 4). Deletion of the N-terminal Strep tag and insertion of the C-terminal stop codon was achieved with QuikChange Site-Directed Mutagenesis Lightning kit (Agilent). Mutations E277Q, Y463H, S257A, S257W, K444R, K444Q, and K444E were generated using QuikChange Site-Directed Mutagenesis Lightning kit (Agilent). *E.coli* strains were cultured in 2x YT (Fisher BioReagents) liquid media or 2x YT agar at 37 °C. Sequences of oligonucleotides used for generation of plasmids and mutants are listed in Supplementary Table 5.

### Expression and purification of BthA

BthA was co-transformed with a plasmid containing cytochrome *c* maturation cassette^42^, into *E. coli* BL21(DE3) competent cells. Starter cultures of 5 mL were grown overnight at 37 °C in 2x YT, supplemented with 100 µg mL^−1^ ampicillin and 35 µg mL^−1^ chloramphenicol. The culture was harvested and resuspended in fresh media before inoculating 1 L flask of 2x YT for bulk expression at 37 °C with 220 rpm until OD_600_ = 0.8 was reached. Cells were cooled and induced with 100 µM β-d-thiogalactopyranoside (IPTG, Bio-rad) for 17 h at room temperature with shaking at 150 rpm.

Cells were harvested, resuspended in lysis buffer (50 mM HEPES, 300 mM NaCl, 5% glycerol, pH 7.8, 1 mM phenylmethanesulfonyl (PMSF)), and were lysed by sonication. Clarified lysate was obtained after centrifugation at 18,000 × *g* for 35 min Supernatant was diluted with Buffer A (50 mM HEPES, 50 mM NaCl, pH 7.8) and loaded onto SP-Sepharose cation exchange resin (GE Healthcare). Resin was washed with Buffer A, and eluted with 50% of Buffer B (50 mM HEPES, 500 mM NaCl, pH 7.8). Protein eluted from SP-sepharose was pooled and concentrated to 1 mL before loading onto S-200 size exclusion column (GE Healthcare) at 4 °C. Purity of BthA containing fractions was assessed by 12.5% sodium dodecyl sulfate polyacrylamide gel electrophoresis. Pure fractions were concentrated in Amicon 30K MWCO and exchanged into storage buffer (50 mM HEPES, 100 mM NaCl, 10% glycerol, pH 7.8) before being stored at −80 °C. Protein concentration was determined by Bradford assay.

### Ultraviolet (UV)–Visible spectroscopic analysis

UV–Visible spectra for the oxidized and reduced state of BthA were obtained on a Cary50 spectrophotometer (Agilent) using 1 mL quartz cuvettes (Starna cells). The as-purified, oxidized state was collected under aerobic conditions at 21.0 °C in 50 mM HEPES, 50 mM NaCl, pH 7.8 buffer. For reduction of the heme centers, either sodium dithionite or sodium ascorbate was added to the cuvettes to a final concentration of 1 or 5 mM under anaerobic conditions with the use of gas-tight syringes (Hamilton). CO binding to the reduced form of the protein was achieved by bubbling in CO for 30 s before recording the spectrum.

### Steady-state kinetic assay

Peroxidase activity was determined using 2,2'-azino-bis(3-ethylbenzothiazoline-6-sulfonic acid) (ABTS, Sigma) as the artificial electron donor. Assay was performed aerobically in assay buffer (50 mM phosphate, pH 6.5) at 21.0 °C, using H_2_O_2_ concentrations ranging from 0.3 to 30 µM. ABTS and H_2_O_2_ were reacted for 10 s before addition of enzyme, and background reactivity was subtracted from each trace. Enzyme was added to final concentration of 30 nM (determined by Bradford). The increase of absorbance at 420 nm was monitored with extinction coefficient *ε*_420_ = 36 mM^−1^ cm^−1^. Data were recorded on a Cary50 spectrophotometer (Agilent).

### Potentiometric titration

Redox titrations were performed anaerobically with an optically transparent thin-layer electrochemical (OTTLE) cell. The OTTLE cell set-up utilized 3D printing to construct a cuvette spacer that allowed for small sample volume, as described by Brisdendine et al.^25^. The spacer was printed by Stratasys using polyjet amber clear material. The cell consisted of a working electrode (0.25 mM thick gold foil and 0.5 mM gold wire (Aldrich)), a M-401 Ag/AgCl reference electrode (Microelectrodes, Inc.) and a 1 mM thick platinum wire as a counter electrode. The spacer assembled with the three electrodes mentioned above was placed in a 10 mM quartz cuvette (Starna Cells). A Teflon cap was fit to the cuvette top equipped with a port for the reference, counter, and working electrode that allowed for placement of each electrode in their respective compartment before the cap was sealed and the cuvette purged with argon. The three-component electrode system was set-up to a PGSTAT 12 AutoLab (Ecochemie) potentiostat. The UV–Visible spectrum was obtained on a Cary50 spectrophotometer after the system reached equilibration at the given potential. Multicomponent buffer (5 mM HEPES, MES, MOPS, CAPS, TAPS, CHES, 100 mM NaCl, pH 7.0) was purged sufficiently with argon before use to remove oxygen. Mediators ranged from −80 to −290 mV vs. SHE and included potassium indigo-trisulfonate, Safranin-T, FMN, and Janus Green B.

### Protein film voltammetry

Electrochemistry experiments were performed using a three-component electrode system in an anaerobic chamber (MBraun). Set-up consisted of a saturated calomel electrode (Fisher) and platinum wire counter electrode. Pyrolytic graphite edge (PGE) electrodes were used as the working electrode. To prepare the PGE electrode, the graphite surface was sanded and polished with an aqueous mix of 1 µM alumina before sonication in Milli-Q water for 2 min Polished electrodes were then left to dry before depositing 16 µL of multi-walled carbon nanotube (MWCNT) mixture. Mixture consisted of 1 mg MWCNT (sigma) in 1 mL of dimethylformamide (DMF). Electrodes were left to dry overnight to ensure surface was sufficiently coated and DMF was no longer present. Before depositing protein, electrodes were rinsed excessively with water and left to dry. For preparation of adsorbed protein onto the MWCNT-coated surface, 1–4 µL of ~400 µM protein in 50 mM HEPES, 100 mM NaCl, 10% glycerol, pH 7.8 was pipetted onto the electrode and left to stand for 1 min before excess protein was pipetted off. Cell solution consisted of multicomponent buffer [5 mM HEPES, MES, MOPS, CAPS, TAPS and CHES, 100 mM NaCl] to allow for collection at varying pH from 5.0 to 9.5. Experiments were carried out at 21 °C. Measurements were recorded on a PGSTAT 12 Autolab (Ecochemie) potentiostat. Data were analyzed using QSoas^43^.

### Near-infrared spectroscopic analysis

The spectra for diferric and H_2_O_2_ treated BthA were recorded on a Shimadzu UV-3600 spectrophotometer in a 1 mL quartz cuvette (Starna Cells). H_2_O_2_ concentrations used for near-stoichiometric additions to BthA were determined using the extinction coefficient *ε*_240 nm_ = 43.6 M^−1^ cm^−1^. For Y463H, E277Q, S257W, S257A, and K444R variants, H_2_O_2_ was added to either one or ten equivalents. Spectra were obtained aerobically at room temperature in 50 mM HEPES, pH 7.8 buffer. For S257A, S257W, and K444R spectra were recorded in 50 mM HEPES, 100 mM NaCl, 10% glycerol, pH 7.8.

### EPR and Mössbauer spectroscopy

Incorporation of ^57^Fe was achieved through expression of BthA in minimal media. Spizizen’s minimal media (MM)^44^ was prepared for expression of ^57^Fe-BthA. Media consisted of 15 mM (NH_4_)_2_SO_4_, 80 mM K_2_HPO_4_, 44 mM KH_2_PO_4_, and 4 mM sodium citrate. For each 1 L of autoclaved MM, glucose was added to a final concentration of 0.4% and 10 mL of trace metal solution consisting of 0.6% MgCl_2_, 0.05% CaCl_2_, 0.006% MnCl_2_, 0.02% ZnCl_2_, 0.003% CuCl_2_, 0.003% CoCl_2,_ and 0.005% Na_2_MoO_4_ was added aseptically. The quantity of background ^56^Fe was determined using ferrozine iron assay^45^ for each flask of media before inoculation. Background ^56^Fe was determined to be 0.02 ng L^−1^. ^57^Fe metal (Cambridge Isotopes) was added to a final concentration of 2 mg L^−1^. Expression of ^57^Fe-BthA was carried out as described above with the final ^57^Fe-BthA sample prepared in the same final sample buffer (vide infra). X-band EPR spectra and Mössbauer data were recorded as reported recently^46^. Briefly, EPR spectra were recorded on a Bruker Elexsys spectrometer equipped with an Oxford ESR 910 cryostat and a Bruker bimodal cavity using a modulation amplitude and frequency of 1 mT and 100 kHz for all spectra. The microwave frequency was calibrated with a frequency counter and the magnetic field was calibrated with a calibrated Hall probe. The temperature was calibrated with a carbon glass resistor (Lakeshore CGR-1-1000) mounted inside an EPR tube. EPR signals were quantified relative to a 1 mM Cu(II)EDTA standard in 10% glycerol. The Cu(II) concentration was quantified by inductively coupled plasma mass spectrometry. Concentrations of spins were determined with the software SpinCount developed by M.P. Hendrich^47^. The software diagonalizes the spin Hamiltonian,

1 $$H = \beta _{\mathrm{e}}B \cdot g_{\mathrm{e}} \cdot S + S \cdot D \cdot S,$$

where all parameters have their usual definitions. The quantitative simulations are least-squares fits of the experimental spectra that accounts for all instrumental intensity factors, which allows the simulated spectra to be assigned a precise sample concentration.

^57^Fe Mössbauer spectra were recorded with two spectrometers using Janis Research dewars. All isomer shifts are reported relative to Fe metal at 298 K. The simulations of Mössbauer spectra were also fit using SpinCount, employing the spin Hamiltonian:^47^

2 $$H = \hskip 3pt 	 \beta _{\mathrm{e}}B \cdot g_{\mathrm{e}} \cdot S + D \left[ {\mathrm{Sz}}^2-S^2 + E/D\left( {S_{\mathrm{x}}^2 + S_{\mathrm{y}}^2} \right) + S \cdot A \cdot I-g_{\mathrm{n}}\beta _{\mathrm{n}}B \cdot I \right. \\ \hskip 10pt 	+ \left[3I_{\mathrm{z}}^2-I^2 + {\mathrm{\eta }}\left[ {I_{\mathrm{x}}^2-I_{\mathrm{y}}^2} \right] \right]$$

### Crystallization of BthA

Initial crystallization screening was performed using the JCSG + suite (Qiagen), and all optimization and cryoprotectant solutions were prepared using chemicals purchased from Hampton Research. Crystal conditions for BthA were identified through sparse matrix screening at 21 °C using a Crystal Phoenix (Art Robbins Instruments). Initials crystals formed from 150 nL of BthA (12 mg mL^−1^ in 50 mM HEPES, pH 7.8, 100 mM NaCl, 5% (v/v) glycerol) that was added to 150 nL of precipitant solution (200 mM ammonium nitrate, 20% (w/v) PEG 3350) and equilibrated against a reservoir of 70 μL precipitant solution using the sitting drop method. After 3 weeks, crystals grew out of a heavy precipitate to form red rod-like crystals, which were used to generate a microseed stock in precipitant solution for further optimization.

Through a combination of microseeding and precipitant solution optimization, larger red rod-like crystals were grown by adding 1 μL BthA (12 mg mL^−1^ in 50 mM HEPES, pH 7.8, 100 mM sodium chloride, 5% (v/v) glycerol) to 0.8 μL precipitant solution (300 mM ammonium nitrate, 22% (w/v) PEG 3350) and 0.2 μL microseed stock and equilibrating against 500 μL precipitant solution using the sitting drop method. After 3 weeks, crystals grew out of a heavy precipitate. Crystals were harvested and cryoprotected in a solution of 200 mM ammonium nitrate, 25% PEG 3350, and 10% glycerol for several minutes and flash-frozen in liquid nitrogen.

### Data collection and processing

A native data set of Wt BthA was collected at a wavelength of 0.9791 Å and temperature of 100 K on a Pilatus 6M pixel detector on beamline 24-ID-C at the Advanced Photon Source (Argonne, IL). The data were indexed, integrated, and scaled in spacegroup P2_1_2_1_2_1_ with cell edges *a* = 51.20 Å, *b* = 84.66 Å, and *c* = 95.80 Å to 1.54-Å resolution using HKL2000^48^.

### Model building and refinement

The structure of BthA was determined to 1.54-Å resolution by molecular replacement in PHASER^49^. The structure of MauG (PDB: 3L4M, 34% sequence identity)^7^ was used as a search model after pruning to the last common side-chain heteroatom with phenix.sculptor^50^. A solution with 1 molecule per asymmetric unit was found with LLG and TFZ scores of 201 and 16.9, respectively. The initial model had values of 53.0% and 53.6% for the working and free *R*-factors, respectively. Iterative rounds of model building and refinement were performed in COOT^51^ and phenix.refine^52^, respectively. Heme cofactors were added using a *c*-type heme cif file from the CCP4 database, which was modified to reduce planarity restraints, allowing for heme ruffling^53^, and a parameter file was used to covalently link the two heme cofactors to C188 and C191 as well as C367 and C370. Once the polypeptide chain and heme cofactors were built, waters were added automatically and curated manually. Riding hydrogen atoms were added to the protein model using phenix.ready_set^52^. Later refinements and the final structure were verified using composite omit maps. The final model contains residues P126-Q242 and L247-P543 of 543, two covalently bonded *c*-type heme cofactors, five nitrate ions, two chloride ions, and one glycerol molecule. The first 59 residues of the construct are not modeled. Analysis of the final model was performed with MolProbity^54^, identifying 96.33%, 3.67%, and 0% of residues in the favored, allowed, and disallowed regions of the Ramachandran plot. Rotamer analysis shows 0.61% rotamer outliers. M368, and E379 are listed as rotamer outliers, but their sidechains show clear electron density. The data collection, processing, and refinement statistics are summarized in Supplementary Table 3. Figures were generated using PyMOL (Schrödinger, LLC). All structural software packages were compiled by SBGrid^55^.

### Calculations of ET pathways of BthA

To evaluate the electron transfer pathways between BthA heme cofactors, the HARLEM program (http://harlem.chem.cmu.edu/index.php) was used for predicting electron transfer characteristics using PATHWAYS^56^. The distance based approach of Dutton^57^ was used to calculate ET parameters, including the relative values of the electron coupling element (*H*_AB_), the decay constant (*β*), and packing (*ρ*). The relevant ET parameters were calculated for both a single-step process from the 6c heme (Donor) to the 5c heme (Acceptor) using both Wt BthA and a computationally generated S257W mutant, as well as for MauG and the appropriate W93S mutant, as in Geng et al.^27^. These calculations were then compared to two-step processes where the Ser257 (or Trp93) position was used a formal redox intermediary.

### Quantitative real-time PCR

*B. thailandensis E264* (Wt Bt) was adapted to anaerobic or aerobic growth for at least 30 generations prior to RNA isolation. Cells were routinely grown at 37 °C in Luria-Broth. Anaerobic cultures were set-up in sealed Balch tubes with 75% N_2_, 20% CO_2_, and 5% H_2_ at 5 psi over ambient pressure and supplemented with 10 mM sodium nitrate as the electron acceptor. RNA was extracted from triplicate cultures grown to stationary phase. Briefly, cells were harvested and resuspended in 1 mL RNApro solution (MP Biomedical). Cell lysate was generated by sonication using a micro-sonicator for 10 s (Bronson). Lysate was centrifuged for 5 min at 13,000 rpm, 4 °C. Supernatant was transferred to a new tube and incubated at room temperature for 5 min before addition of 300 μL chloroform. Sample was transferred to a 2 mL pre-spun phase-lock tube (QuantaBio) and incubated for 5 min before centrifugation at 13,000 rpm. Upper phase was mixed with 500 μL of 100% RNase free ethanol and incubated at 20 °C for 1 h. RNA was pelleted at 13,000 rpm, 4 °C for 15 min and washed with 500 μL of 75% EtOH and let to dry before resuspension in 100 μL nuclease-free H_2_O (Invitrogen). Partially purified RNA was cleaned up using the RNeasy Mini Kit (Qiagen) following manufacturer’s recommendation. RNA concentrations were quantified and placed at −80 °C until needed. Complementary DNA (cDNA) was synthesized using TaqManR reverse transcription reagents (Applied Biosystems) following manufacturer’s protocol. Quantitative PCR analysis was carried out using 2X SYBR Green ROX PCR master mix (ThermoFisher) in a reaction of 100 ng template cDNA, and 0.3 µM reverse and forward primers for each gene were analyzed in a total volume of 20 µL. Primers used for amplification of *bthA* (bthA-F, bthA-R), *phosA* (phosA-F, phosA-R), and housekeeping gene *gyrB (gyrB-F, gyrB-R)* are listed in Supplementary Table 5. Control gene *gyrB* was selected based on previous reports for similar experiments in *B. thailandensis*^58^. Reactions were carried out on a 7900ht instrument (Applied Biosystems). Relative quantification of gene expression by qPCR was determined by use of the 2^∆∆Ct^ method^59^.

## Supplementary information

Supplementary Information

Reporting Summary

## Data Availability

The sequences used in the SSN are from the Uniprot database, with accession codes listed in Supplementary Table 1. Crystallographic data has been deposited with accession code 6NX0.pdb at the Protein Data Bank. Other data are available from the corresponding author upon reasonable request.
