# Supplementary material for: A widely distributed diheme enzyme from Burkholderia that displays an atypically stable bis-Fe(IV) state
Source: Nat Commun. 2019 Mar 7;10:1101. doi: 10.1038/s41467-019-09020-4 (PMC6405878; doi:10.1038/s41467-019-09020-4)
Supplement: Supplementary file 1 — Supplementary Information [file 41467_2019_9020_MOESM1_ESM.pdf]

Supplementary Information

A widely distributed diheme enzyme from *Burkholderia* that displays an atypically stable *bis*-Fe(IV) state

K.Rizzolo, *et al.*

Supplementary Table 1 I List of Proteins from Cluster IIIb of CCP-MauG SSN (Figure 1)

Uniprot ID of Represented Protein	Sequences with 80% ID to Represented Protein	Protein Description	Organism of Represented Protein
A0A0D6MWK0_ACEA	1	Cytochrome c peroxidase/methylamine utilization protein MauG	*Acetobacter aceti NBRC 14818.*
A0A0D6N3U1_9PRO	11	Cytochrome c peroxidase/methylamine utilization protein MauG	*Acetobacter cibinongensis 4H-1.*
A0A0D6N6R1_9PRO	1	Cytochrome c peroxidase/methylamine utilization protein MauG	*Acetobacter cibinongensis 4H-1.*
A0A0D6NC10_9PRO	1	Cytochrome c peroxidase/methylamine utilization protein MauG	*Acetobacter indonesiensis 5H-1.*
A0A0D6NJQ1_9PRO	1	Cytochrome c peroxidase/methylamine utilization protein MauG	*Acetobacter orientalis 21F-2.*
A0A149U3E6_9PRO	6	Methylamine utilization protein MauG	*Acetobacter senegalensis.*
C1F330_ACIC5	1	Di-heme cytochrome c peroxidase family protein	*Acidobacterium capsulatum*
A0A0D6PDH8_9PRO	1	Cytochrome c peroxidase/methylamine utilization protein MauG	*Acidocella aminolytica 101 = DSM 11237.*
K5ZFG9_9PROT	1	Di-heme cytochrome c peroxidase	*Acidocella sp. MX-AZ02.*
A0A023D3H4_ACIM	1	Cytochrome c peroxidase/methylamine utilization protein MauG	*Acidomonas methanolica NBRC 104435.*
F0Q4Y4_ACIAP	1	Di-heme cytochrome c peroxidase	*Acidovorax avenae*
F0QAT2_ACIAP	1	Di-heme cytochrome c peroxidase	*Acidovorax avenae*
A0A109CSF0_AGRV	2	Cytochrome C peroxidase	*Agrobacterium vitis*
A0A0N0GLI5_9NEI	1	Methylamine utilization protein MauG	*Amantichitinum ursilacus.*
A0A0N0XLQ4_9NEI	1	Methylamine utilization protein MauG	*Amantichitinum ursilacus.*
A0A0A0DZ07_9BUR	1	Uncharacterized protein	*Aquabacterium sp. NJ1.*
A0A0F3KTY3_9NEI	1	Cytochrome C peroxidase	*Aquitalea magnusonii.*
A0A060QK26_9PRO	2	Methylamine utilization protein mauG	*Asaia platycodi SF2.1.*
B2IBY9_BEII9	1	Di-haem cytochrome c peroxidase	*Beijerinckia indica subsp. indica*
A0A0N1LDS2_9PRO	1	Cytochrome C peroxidase	*beta proteobacterium AAP99.*
A0A157L0J1_9BOR	1	Methylamine utilization protein MauG	*Bordetella ansorpii.*
A0A157S5C2_9BOR	1	Methylamine utilization	*Bordetella ansorpii.*

		protein MauG	
A0A0S1Y7M8_9BOR	1	Cytochrome C peroxidase	*Bordetella sp. N.*
A0A0E4BLV6_9BRA	13	Putative methylamine utilization protein	*Bradyrhizobium diazoefficiens.*
A0A109K5R8_9BRA	2	Cytochrome C peroxidase	*Bradyrhizobium sp. BR 10303.*
H0TPH0_9BRAD	1	Uncharacterized protein	*Bradyrhizobium sp. STM 3843.*
A0A109EDF1_9BUR	85	Cytochrome B6	*Burkholderia cenocepacia.*
A0A104JEQ1_BURG	3	Cytochrome-c peroxidase	*Burkholderia gladioli*
A0A124UDP2_BURG	1	Cytochrome-c peroxidase	*Burkholderia gladioli*
C5A8I0_BURGB	3	Di-haem cytochrome c peroxidase	*Burkholderia glumae (strain BGR1)*
A0A149PUI4_9BUR	1	Cytochrome-c peroxidase	*Burkholderia monticola.*
A0A0H3KDX0_BURM	56	Cytochrome c peroxidase	*Burkholderia multivorans*
A0A0H3KUU2_BURM	69	Cytochrome c peroxidase	*Burkholderia multivorans*
B9B7J8_9BURK	10	Di-heme cytochrome c peroxidase	*Burkholderia multivorans CGD1*
A8EKP1_BURPE	35	Di-haem Cytochrome c peroxidase family protein	*Burkholderia pseudomallei 406e*
A0A104NQQ1_BURP	1	Cytochrome-c peroxidase	*Burkholderia pyrrocinia*
D5WLT6_BURSC	1	Di-hem cytochrome c peroxidase	*Burkholderia sp. (strain CCGE1002)*
E8YKH7_9BURK	4	Di-heme cytochrome c peroxidase	*Burkholderia sp. CCGE1001*
B5WFS1_9BURK	1	Di-heme cytochrome c peroxidase	*Burkholderia sp. H160.*
B5WQ61_9BURK	1	Di-heme cytochrome c peroxidase	*Burkholderia sp. H160.*
A0A0Q5P3D2_9BUR	3	Cytochrome C peroxidase	*Burkholderia sp. Leaf177.*
A0A0Q5P3K4_9BUR	5	Cytochrome C peroxidase	*Burkholderia sp. Leaf177.*
A0A060P5A7_9BUR	22	Di-heme cytochrome c peroxidase	*Burkholderia sp. RPE67.*
A0A060P964_9BUR	6	Putative cytochrome c peroxidase	*Burkholderia sp. RPE67.*
A0A103DWF2_9BUR	1	Cytochrome-c peroxidase	*Burkholderia sp. TSV85.*
Q2T6B0_BURTA BthA	51	Cytochrome c family protein	*Burkholderia thailandensis*
A0A104RBB5_9BUR	24	Cytochrome-c peroxidase	*Burkholderia ubonensis.*
Q13JY9_BURXL	3	Putative cytochrome c peroxidase	*Burkholderia xenovorans (strain LB400)*
Q13W88_BURXL	4	Putative Di-haem cytochrome c peroxidase	*Burkholderia xenovorans (strain LB400)*
A0A0F0FD14_9BUR	3	Cytochrome C peroxidase	*Burkholderiaceae bacterium 16.*
A0A0F0FEE1_9BUR	4	Cytochrome B6	*Burkholderiaceae bacterium 16.*
A0A0L0MAG6_9BUR	1	Methylamine utilization protein mauG	*Candidatus Burkholderia verschuerenii.*

A0A0L0M7K8_9BUR	1	Cytochrome c peroxidase	*Candidatus Paraburkholderia schumannianae.*
Q7NSA0_CHRVO	3	Uncharacterized protein lCytochrome C peroxidase	*Chromobacterium violaceum*
A0A0A1FG20_9BUR	3	Methylamine utilization protein mauG	*Collimonas arenae*
A0A0A1F439_9BUR	3	Methylamine utilization protein mauG	*Collimonas arenae.*
A0A0A1F463_9BUR	1	Methylamine utilization protein mauG	*Collimonas arenae.*
A0A127QD26_9BUR	1	Di-heme cytochrome c peroxidase family protein	*Collimonas arenae.*
A0A127P5G6_9BUR	5	Cytochrome c family protein	*Collimonas fungivorans.*
A0A127QBC7_9BUR	3	Cytochrome c family protein lCytochrome c peroxidase	*Collimonas pratensis.*
A0A127QU57_9BUR	4	Cytochrome c family protein	*Collimonas pratensis.*
D0IYM5_COMT2	1	Di-heme cytochrome c peroxidase	*Comamonas testosteroni (strain CNB-2)*
H1S6U9_9BURK	1	Cytochrome c peroxidase	*Cupriavidus basilensis OR16.*
H1SF75_9BURK	1	Di-heme cytochrome c peroxidase	*Cupriavidus basilensis OR16.*
Q1LCG2_CUPMC	3	Di-heme cytochrome c peroxidase	*Cupriavidus metallidurans*
A0A132HAZ6_9BUR	3	Methylamine utilization protein MauG	*Cupriavidus metallidurans.*
A0A069ICH9_9BUR	1	Cytochrome C peroxidase	*Cupriavidus sp. SK-3.*
A0A022G295_9BUR	1	Diacylglycerol kinase	*Cupriavidus sp. SK-4.*
A0A059IPA0_9RHO	1	Cytochrome C peroxidase	*Defluviimonas sp. 20V17.*
K2IG95_9GAMM	1	Di-heme cytochrome C peroxidase	*Gallaecimonas xiamenensis 3-C-1.*
A9HNB2_GLUDA	1	Di-heme cytochrome c peroxidase	*Gluconacetobacter diazotrophicus*
F3S881_9PROT	4	Methylamine utilization protein mauG	*Gluconacetobacter sp. SXCC-1.*
G6XL93_9PROT	1	Putative di-heme cytochrome c peroxidase	*Gluconobacter morbifer G707.*
Q5FS76_GLUOX	5	Putative di-haem cytochrome c peroxidase	*Gluconobacter oxydans (strain 621H)*
M9MLY8_GLUTH	9	Di-heme cytochrome c peroxidaseperoxidase/methylamine utilization protein MauG	*Gluconobacter thailandicus NBRC 3255.*
A0A0K2BCF9_9BUR	2	Cytochrome C peroxidase	*Herbaspirillum hiltneri N3.*
A0A0C2BUW6_9BUR	1	Cytochrome C peroxidase	*Herbaspirillum sp. TSA66.*
A0A0A0D6J4_9PRO	1	Cytochrome C peroxidase	*Inquilinus limosus MP06.*
L9PA62_9BURK	1	Methylamine utilization protein mauG	*Janthinobacterium sp. HH01.*
L9PNT8_9BURK	1	Di-heme cytochrome c peroxidase	*Janthinobacterium sp. HH01.*

A0A0M2WE15_9BUR	4	Methylamine utilization protein MauG	*Janthinobacterium sp. KBS0711.*
A0A0M2WL29_9BUR	1	Methylamine utilization protein MauG	*Janthinobacterium sp. KBS0711.*
A0A0D6PZR2_KOME	3	Cytochrome c peroxidase/methylamine utilization protein MauG	*Komagataeibacter europaeus NBRC 3261.*
G2I3E1_KOMMN	1	Methylamine utilization protein MauG	*Komagataeibacter medellinensis*
A0A0L8B0L9_9BUR	1	Uncharacterized protein	*Limnohabitans planktonicus II-D5.*
A0A0N7J9Y7_9BUR	1	Methylamine utilization protein MauG	*Limnohabitans sp. 103DPR2.*
A0A0Q7XTP1_9BUR	1	Cytochrome B6	*Massilia sp. Root1485.*
A0A0Q6TR34_9BUR	1	Cytochrome C peroxidase	*Massilia sp. Root335.*
G6YIR1_9RHIZ	1	Di-heme cytochrome c peroxidase	*Mesorhizobium amorphae CCNWGS0123.*
A0A090E4R3_9RHI	9	Di-heme cytochrome c peroxidase	*Mesorhizobium sp. ORS3324.*
A0A0Q6MTS8_9RHI	2	Cytochrome C peroxidase	*Mesorhizobium sp. Root102.*
A0A0Q8KKP4_9RHI	2	Cytochrome C peroxidase	*Mesorhizobium sp. Root172.*
B8EKA3_METSB	1	Di-heme cytochrome c peroxidase	*Methylocella silvestris*
J7Q4U5_METSZ	1	Di-heme cytochrome c peroxidase	*Methylocystis sp. (strain SC2)*
E6PV15_9ZZZZ	1	Putative Cytochrome-c peroxidase	*mine drainage metagenome.*
F1W1P5_9BURK	1	Putative lipopro transmembrane	*Oxalobacteraceae bacterium IMCC9480.*
A0A0F5K4S6_9BUR	1	Cytochrome C peroxidase	*Paraburkholderia andropogonis.*
A0A0P0RC60_9BUR	5	Cytochrome c peroxidase	*Paraburkholderia caribensis MBA4.*
A0A0D5V466_9BUR	3	Cytochrome c family protein	*Paraburkholderia fungorum.*
A0A0D5VJA9_9BUR	3	Cytochrome c family protein	*Paraburkholderia fungorum.*
A0A069PP74_9BUR	1	Cytochrome C peroxidase	*Paraburkholderia glathei.*
A0A069PPD0_9BUR	2	Cytochrome C peroxidase	*Paraburkholderia glathei.*
A0A069PWJ7_9BUR	1	Cytochrome C peroxidase	*Paraburkholderia glathei.*
B1G872_9BURK	5	Di-haem cytochrome c peroxidase	*Paraburkholderia graminis C4D1M*
B2SZN9_PARPJ	2	Di-haem cytochrome c peroxidase	*Paraburkholderia phytofirmans (strain DSM 17436 / LMG 22146 / PsJN)IParaburkholderia graminis C4D1M.*
A0A0A6SXI5_9BUR	1	Cytochrome C peroxidase	*Paraburkholderia sacchari.*
I5CK89_9BURK	7	Di-heme cytochrome c peroxidase	*Paraburkholderia terrae BS001*
A0A135HVF1_9RHI	1	Cytochrome C	*Paramesorhizobium deserti.*

		peroxidase	
A0A0Q6WL78_9BUR	1	Uncharacterized protein	*Pelomonas sp. Root1237.*
A0A147GSL5_9BUR	1	Cytochrome C peroxidase	*Pseudacidovorax intermedius.*
A0A0Q7TXK6_9RHI	1	Cytochrome C peroxidase	*Pseudolabrys sp. Root1462.*
A0A0S4U2X0_RALS	2	Putative cytochrome c peroxidase signal peptide protein	*Ralstonia solanacearum*
A0A0K1ZSS9_RALS	30	Lipoprotein transmembrane	*Ralstonia solanacearum*
U3GIH0_9RALS	24	Uncharacterized protein	*Ralstonia sp. 5_2_56FAA.*
A0A0B1XXZ0_9RAL	2	Cytochrome C peroxidase	*Ralstonia sp. A12.*
S9T3C6_9RALS	2	Cytochrome B6 lPutative signal peptide protein	*Ralstonia sp. AU12-08.*
A0A117DSL5_9RAL	3	Cytochrome c peroxidase protein	*Ralstonia sp. NT80.*
A0A0Q6M4W4_9BUR	1	Cytochrome C peroxidase	*Rhizobacter sp. Root29.*
J0D0D5_RHILT	10	Cytochrome c peroxidase	*Rhizobium leguminosarum bv. trifolii WSM2297.*
J2WAK9_9RHIZ	6	Cytochrome c peroxidase	*Rhizobium sp. AP16.*
J1T9S4_9RHIZ	1	Cytochrome c peroxidase	*Rhizobium sp. CF142.*
A0A0U3LE31_9BUR	1	Cytochrome C peroxidase	*Roseateles depolymerans.*
A0A031HXI2_9SPH	1	Cytochrome-c peroxidase	*Sphingomonas sp. RIT328.*
A0A0D6MKR3_9PRO	1	Cytochrome c peroxidase transmembrane protein	*Tanticharoenia sakaeratensis NBRC 103193.*
A0A0D6MLR1_9PRO	1	Cytochrome c peroxidase	*Tanticharoenia sakaeratensis NBRC 103193.*
A0A074TA94_9RHO	1	Cytochrome C peroxidase	*Thioclava dalianensis.*
D6CMR6_THIA3	1	Putative Cytochrome-c peroxidase	*Thiomonas arsenitoxydans (strain DSM 22701 / CIP 110005 / 3As)*
A0A0K6HR77_9BUR	3	Cytochrome c peroxidase	*Thiomonas bhubaneswarensis*
A0A0P8ZAA7_VARP	1	Cytochrome B6	*Variovorax paradoxus.*
A0A0P9AKB8_VARP	3	Cytochrome C peroxidase	*Variovorax paradoxus.*
J2KN89_9BURK	7	Cytochrome c peroxidase	*Variovorax sp. CF313.*
Q2SV64_BURTA BthB	38	Cytochrome C peroxidase	*Burkholderia thailandensis*

Proteins listed represent individual nodes in Cluster IIIB. Representative protein is also associated with protein sequences which share 80% or more sequence identity.

Protein	K_M (μM)	k_{cat} (s^{-1})
Wt	3.4 ± 0.4	5.3 ± 1.5
Y463H	1.9 ± 0.9	5.0 ± 1.7
Y463M	3.3 ± 0.4	6.4 ± 0.8
S257A	2.1 ± 0.2	6.0 ± 0.1
S257W	2.2 ± 0.4	3.5 ± 0.3
E277Q	Nd	Nd

Supplementary Table 2 I Kinetic analysis of BthA and its variants as peroxidases. Assays were performed in 50 mM NaH_2PO_4, pH 6.5 with 3 mM ABTS as the electron source, using H_2O_2 concentrations ranging from 0.3 μM to 30 μM and a final concentration of enzyme of 30 nM. Variants show similar kinetic parameters compared to Wt BthA. Kinetic parameters were determined from Michaelis-Menten fit of the data. Parameters represent average of three independent kinetic assays.

Supplementary Table 3 I X-ray crystallographic data collection and refinement statistics

	NT BthA
**Data collection**	
Space group	$P2_12_12_1$
Cell dimensions	
a, *b*, *c* (Å)	51.21, 84.78, 95.83
Resolution (Å)	50.00-1.54
R_{sym}	9.9 (74.5)*
<*I*> / <s*I*>	19.0 (2.22)
Completeness (%)	99.6 (99.0)
Redundancy	7.5 (5.3)
CC1/2	(0.745)
**Refinement**	
Resolution (Å)	47.92-1.54
No. reflections	62344
R_{work} / R_{free}	14.80/17.82
No. atoms	
Protein	6274
Heme	150
Ligand/ion	36
Water	587
B-factors (Å^2)	
Protein	19.36
Heme	11.03
Ligand/ion	35.14
Water	29.28
R.m.s. deviations	
Bond lengths (Å)	0.009
Bond angles (°)	1.092

*Values in parentheses are for highest-resolution shell.

Supplementary Table 4 I Plasmids and gene sequences used in this study

Name	Description	Reference
pETSN	OmpA cleavable periplasmic leader sequence, N-terminal Strep-tag and C-terminal 6x-His tag	Ref 29
pKR2	Insertion of BthA at position S69 into pETSN via EcoRI and XhoI restriction enzyme cut sites	This Study
pKR4	Deletion of N-terminal Strep-tag and C-terminal 6x-His tag from pKR2	This Study
bthA gene codon optimized	gtgaccggtcgtcgccgtagtagtgcagcccgtcgtgcagttcctgcagcagcactgaccgcacgtgca<br>agcgtgaccgcatttgccctgggcccggcagcattaggtaccacacgtacacgtcgtaccgtgctgacc<br>ctgagcaccattgtgcgtgccgcatgtggtgcactggccgcatgtgcaagtgccatcgcattagcaagcg<br>gtccggcagccccggatgcaacccatagcacacatgcagcccgtcctgcaagcgcagcaagcagcac<br>cagtccgatgaatccgacaagtacacctggcgccagcggtccggcacacgcaaaagcagcattagatg<br>ccgcacgcgcaaaagccgcaccgcctagtccgccgacaaccgttctgttaccgggtgccccgccggaa<br>cgcgttgttgataccattggtcgcggcacccctcaggttgccagcaaagttgacccgaccgcagccgtgt<br>ttcgccctgatccgaccctggcagccctgggtaagcgcgtgttcttcgatccggccctgagtgaaccgcg<br>cggtatgagctgtgccagctgccacgatcctggtcgcgcatttgcaccgacactgagcccggccgcact<br>ggccggtccgcgtgtgcctcaaggtagtcgcccgggccattttagccgccgtaatgccccgagcctgct<br>gtatgtgcgctatgttccgcgtcgccatttctatcaggatgacgatgcactggccccggcaccgttcggtg<br>gtctgtttagtgatggtcgcgccgataccctggcagaacagttacgtggcccgctgtttgatcctgatgaga<br>tgaataatgcaagtgccgccgcactgatgcgcaagatcggccgcacaggtttaggtgccgccttagccg<br>gtcgttttggccctagcgttcgccgtgatccggaacgcatggtgcgtgttctgggtgaagccatgcaagcc<br>tatctgcagagtgacgagatggcaccgtttagcagccgttatgacgcatacgtgaccaaacgcgcaccgt<br>taaccccgcaggaaatgcgcggtctggcactgtttcgcaacccggacaaaggtaactgcatgagctgcc<br>acacactgagcgacaccgcaagtcgcccggaacgcagcctgttcaccgatttcggctatgacgccattg<br>ccgttccgcgtaatcgtgccttacctgccaatcgcgatccgcgtcactttgataacggtctgtgcgataccg<br>cagccaaactgcgctggccggaacctacacagtggtgtgcatatctgcgtaccccgggcttacgcaacg<br>tggccatcaaggaaagttttatgcacaatggtgtgttcgataccctgcgtgacgcagtggcattttacaatac<br>ccgcagcaccgatccggcacgctggtatcatggtcgcgatacctttgacgacgtgccgcgtgcctatcgt<br>ggcaatgttaatgtgaacagcaccccgatgaaccgccgccctggtacaccgcctgcaatgaccgatgca<br>gacgtggatgatctggtggcatttctgcgtaccctgacagatgcccgttatgttggtctgatgccgacagcc<br>ccggacggtaaggcagcacgtccttaa	This study

Supplementary Table 5 l Sequences of oligonucleotides used in this study

Name	Sequence (5' —> 3')
pKR2-f	CGAGACCGCGGTCCCGAATTCAAGCGGTCCGGCAGCCCCGG
pKR2-r	CCGGGGCTGCCGGACCGCTTGAATTCGGGACCGCGGTCTCG
pKR3-f	GCTACCGTAGCGCAGGCCGCTAGCAGCGGTCCGGCAGCCCCGGAT
pKR3-r	ATCCGGGGCTGCCGGACCGCTGCTAGCGGCCTGCGCTACGGTAGC
pKR4-f	GACGGTAAGGCAGCACGTCCTTGAGATCCGGCTGCTAACAAAGCC
pKR4-r	GGCTTTGTTAGCAGCCGGATCTCAAGGACGTGCTGCCTTACCGTC
E277Q-F	CGTGGCCCGCTGTTTGATCCTGATCAGATGAATAATGCAAGTGCCGCCGCA
E277Q-R	TGCGGCGGCACTTGCATTATTCATCTGATCAGGATCAAACAGCGGGCCACG
Y463H-F	ACCCTGCGTCAGGCAGTGGCATTTCACAATACCCGCAGCACCGATCCGGCA
Y463H-R	TGCCGGATCGGTGCTGCGGGTATTGTGAAATGCCACTGCGTCACGCAGGGT
Y463M-F	ACCCTGCGTGACGCAGTGGCATTTATGAATACCCGCAGCACCGATCCGGCA
Y463M-R	TGCCGGATCGGTGCTGCGGGTATTCATAAATGCCACTGCGTCACGCAGGGT
S257A-F	GCACCGTTCGGTGGTCTGTTTGCAGATGGTCGCGCCGATACCCTG
S257A-R	CAGGGTATCGGCGCGACCATCTGCAAACAGACCACCGAACGGTGC
S257W-F	GCACCGTTCGGTGGTCTGTTTTGGGATGGTCGCGCCGATACCCTG
S257W-R	CAGGGTATCGGCGCGACCATCCCAAAACAGACCACCGAACGGTGC
K444R-F	GTGGCCATCCGTGAAAGTTTTATGCACAATGGTGTGTTCGATACCCTG
K444R-R	AAAACTTTCACGGATGGCCACGTTGCGTAAGCCCGGGGT
K444E-F	GTGGCCATCGAAGAAAGTTTTATGCACAATGGTGTGTTCGATACCCTG
K444E-R	AAAACTTTCTTCGATGGCCACGTTGCGTAAGCCCGGGGT
K444Q-F	GTGGCCATCCAGGAAAGTTTTATGCACAATGGTGTGTTCGATACCCTG
K444Q-R	AAAACTTTCCTGGATGGCCACGTTGCGTAAGCCCGGGGT
gyrB-F	ATCCGACGATCTTCCACATC
gyrB-R	CAGCACGTTTTCGTTGTAGC
bthA-F	GTACCTGCAAAGCGACGAAATGG
bthA-R	TGTGGCAGCTCATGCAGTTG
phosA-F	CTGCAAAAACAGCCCGTGG
phosA-R	ATCGAGGAAGCGGAACCAG

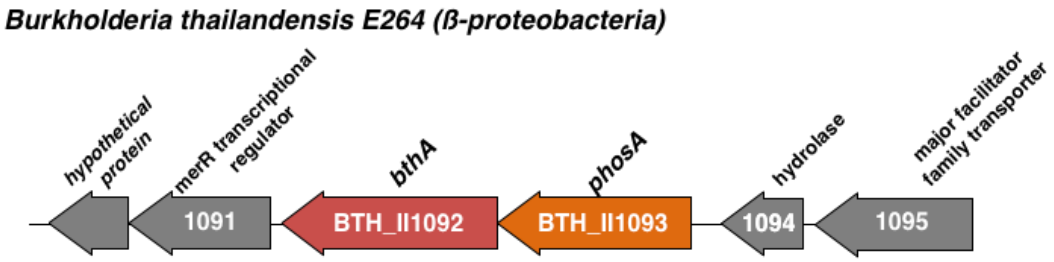

Supplementary Figure 1 l Genomic context of *bthA* in *B. thailandensis.* Putative gene operon of *bthA* (BTH_II1092) in *B.thailandensis E264. bthA* gene (red) is shown downstream of a potential protein partner (BTH_II1093, orange) identified as a putative ser/thr protein phosphatase which we refer to as *phosA*. Other genes shown in the operon are labeled based on annotation of predicted function.

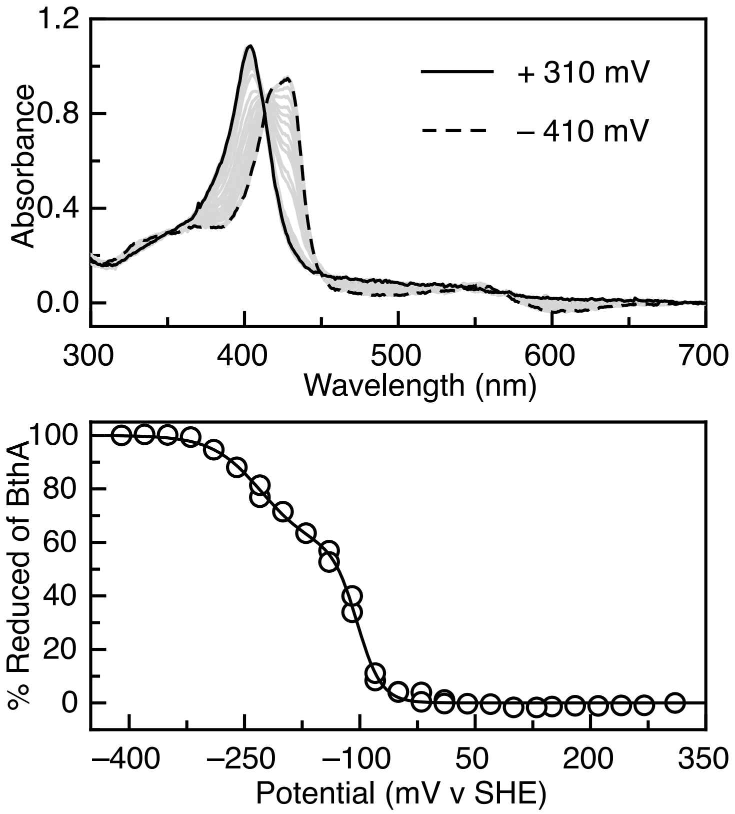

Supplementary Figure 2 l Absorption Spectra of BthA from OTTLE Cell Titration. UV-Vis spectra were recorded for each set potential (top). As BthA was reduced, Soret shifted from 402 nm to 429 nm. Change in absorbance (bottom) was plotted versus recorded potential. Absorption scans were baseline corrected. Titration curve was fit to Nernst equation.

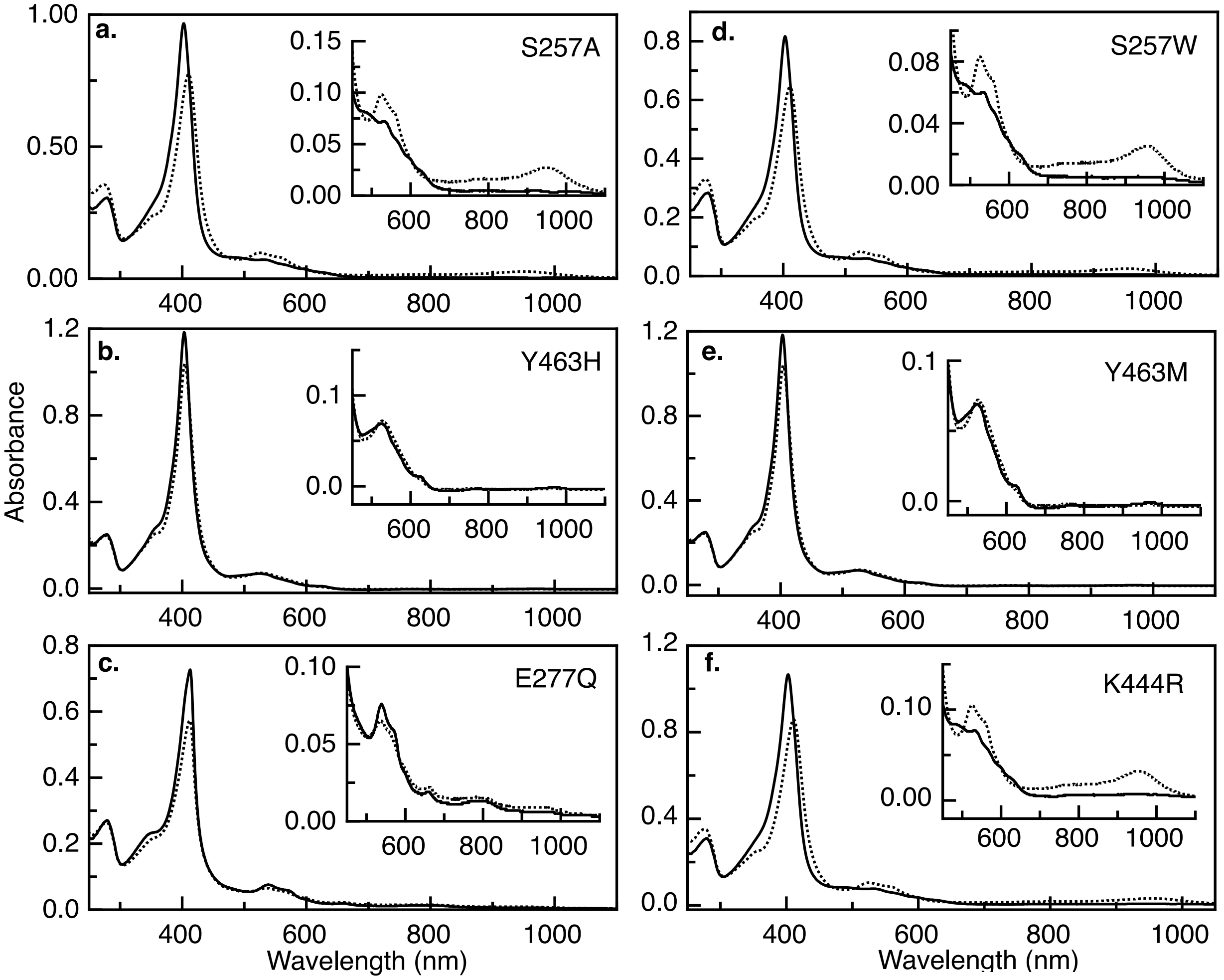

Supplementary Figure 3 l NIR spectra of Variants. Spectra for S257A (a), S257W (d) and K444R (f) were collected in 50 mM HEPES, 100 mM NaCl, 10% glycerol pH 7.8. For Y463H (b), E277Q (c) and Y463M (e) buffer used was 50 mM HEPES, pH 7.8. Protein spectra (5 μM) were collected in the diferric state (solid line) before addition of 10 eq. H_2O_2 (dotted line). K444R (5 μM) produced a spectrum similar to Wt after addition of 1 eq. H_2O_2. Variants Y463H, Y463M and E277Q did not produce a feature at 960 nm as was observed for Wt (Figure 3).

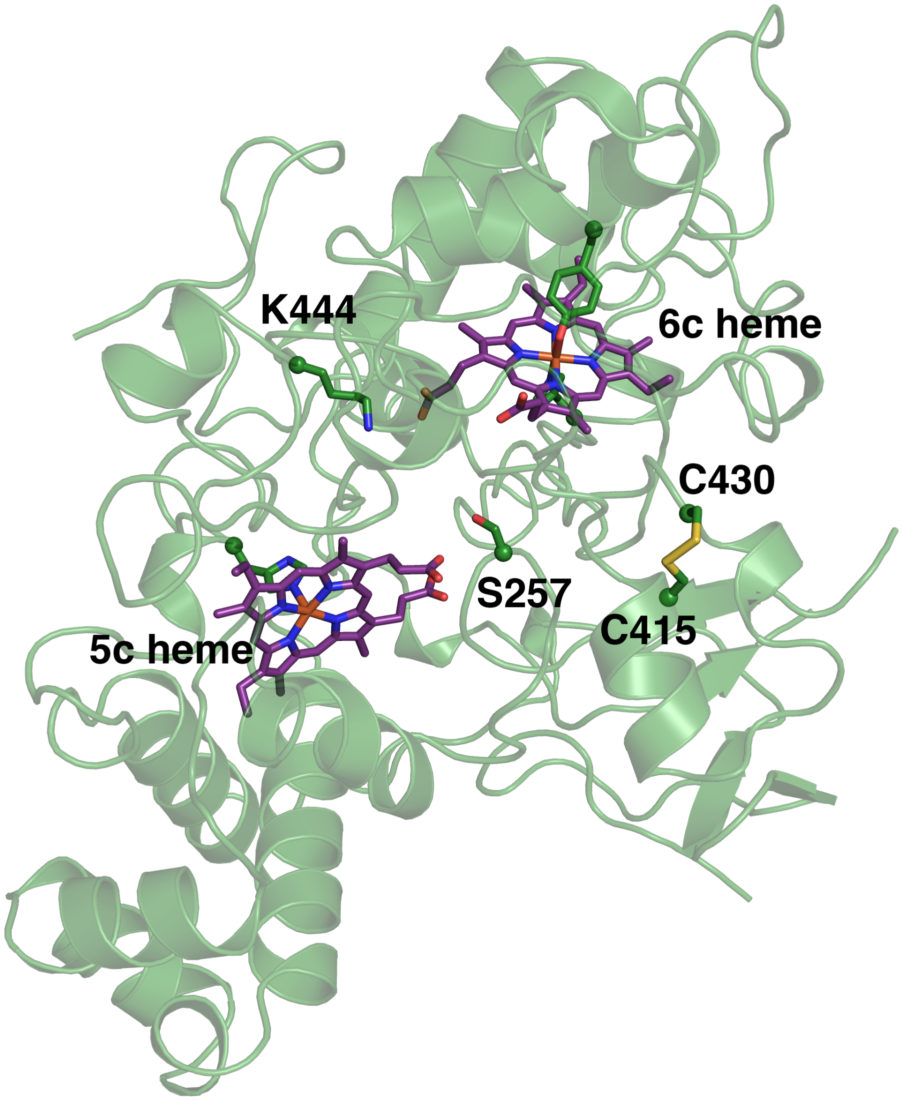

Supplementary Figure 4 | The structure of *B. thailandensis* BthA. Select regions of interest highlighted. Structure represented as in Figure 4.

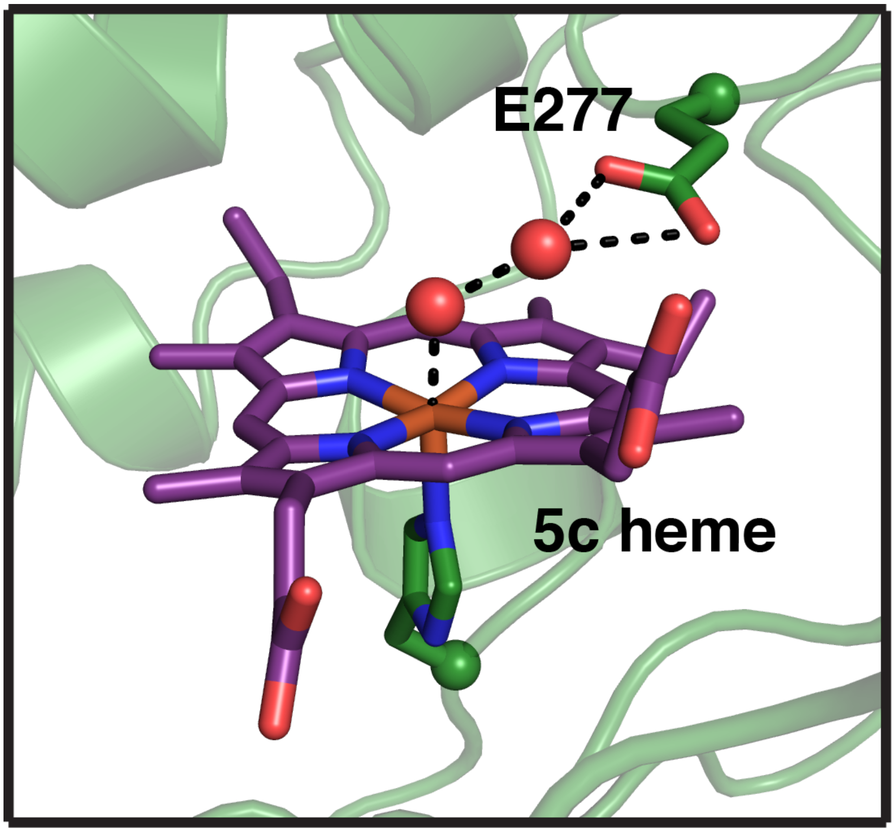

Supplementary Figure 5 l E277 is positioned for acid-base catalysis at the 5c heme. BthA represented as in Figure 4.

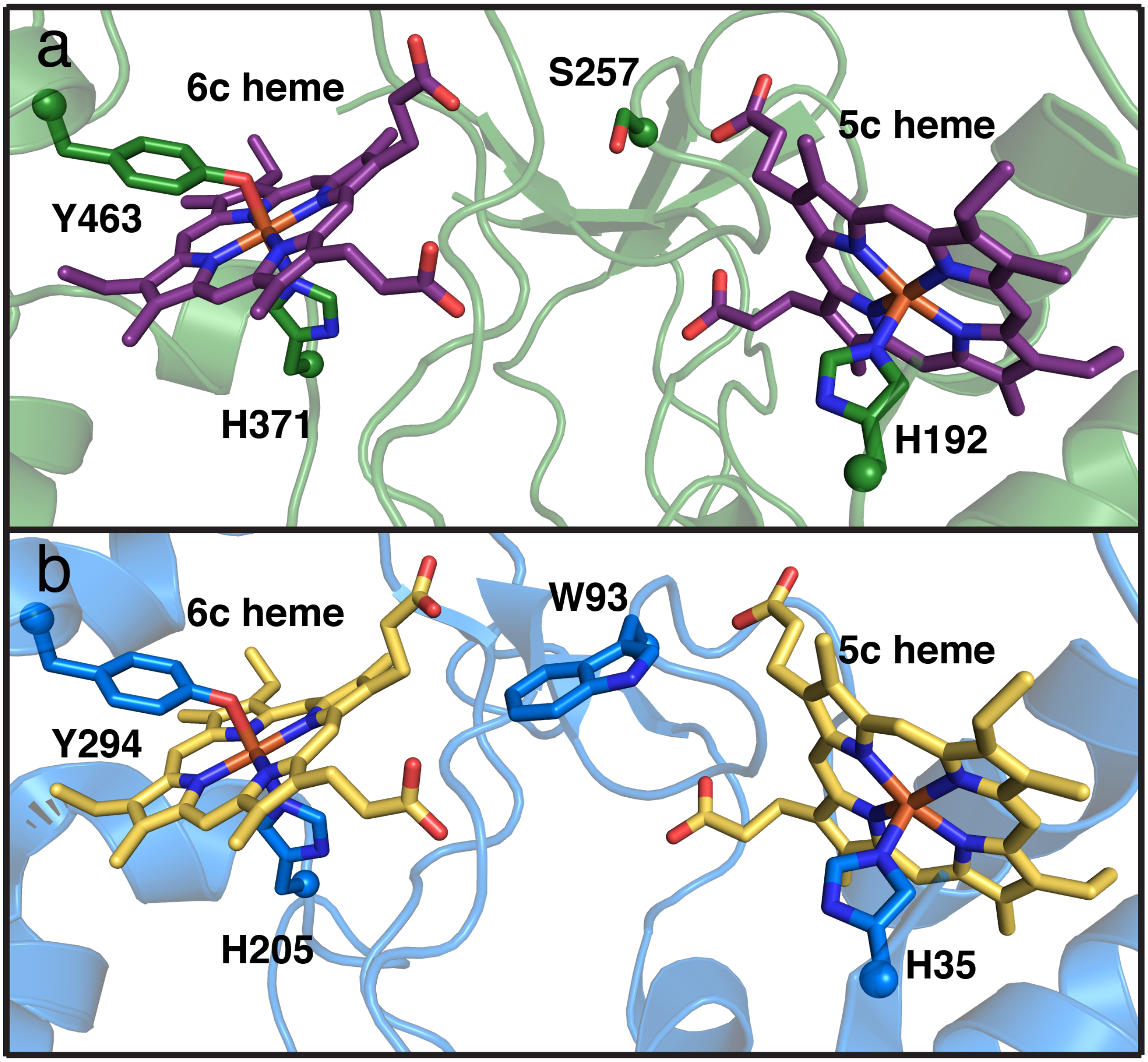

Supplementary Figure 6 I BthA lacks a conserved interheme tryptophan. a) In BthA, as shown in Figure 4a, Ser257 is positioned between the 5c and 6 hemes. b) In MauG, there is a conserved tryptophan residue to mediate interheme electron transfer. BthA represented as in Figure 4. MauG peptide and heme carbon shown in marine and yellow, respectively. MauG peptide backbone shown in cartoon and side chains shown in stick representation.

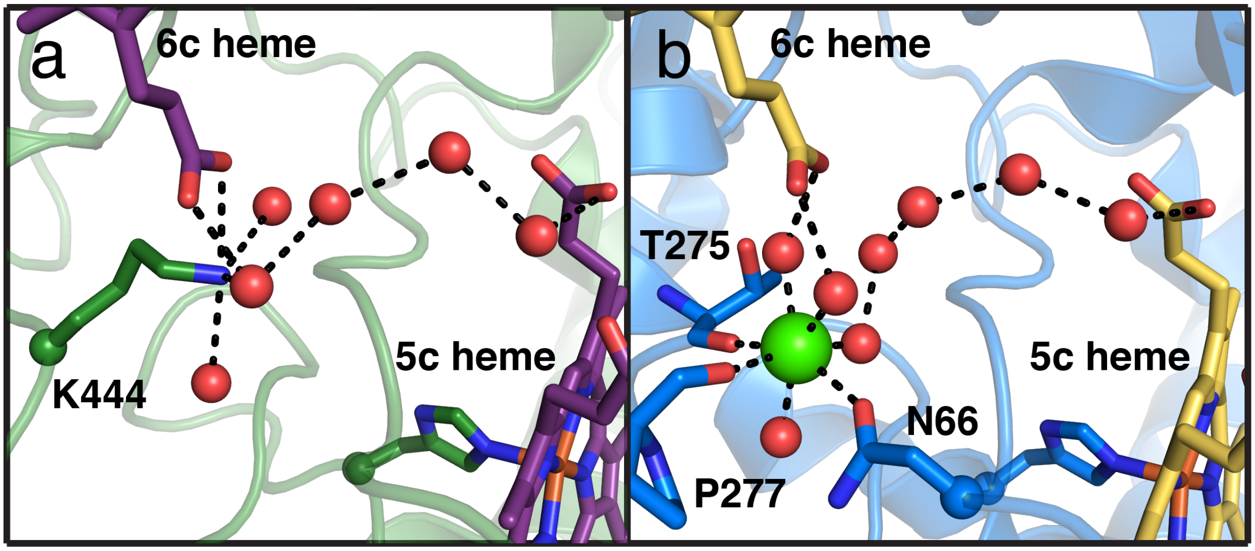

Supplementary Figure 7 | A conserved ordered water network between 5c and 6c hemes. a) In BthA K444 mediates an ordered water network between the two heme propionate groups. b) In MauG (PDB 3L4M), a protein-coordinated calcium mediates a similar network. BthA represented as in Figure 4. MauG respresented as in Figure S6. Calcium shown in light green.

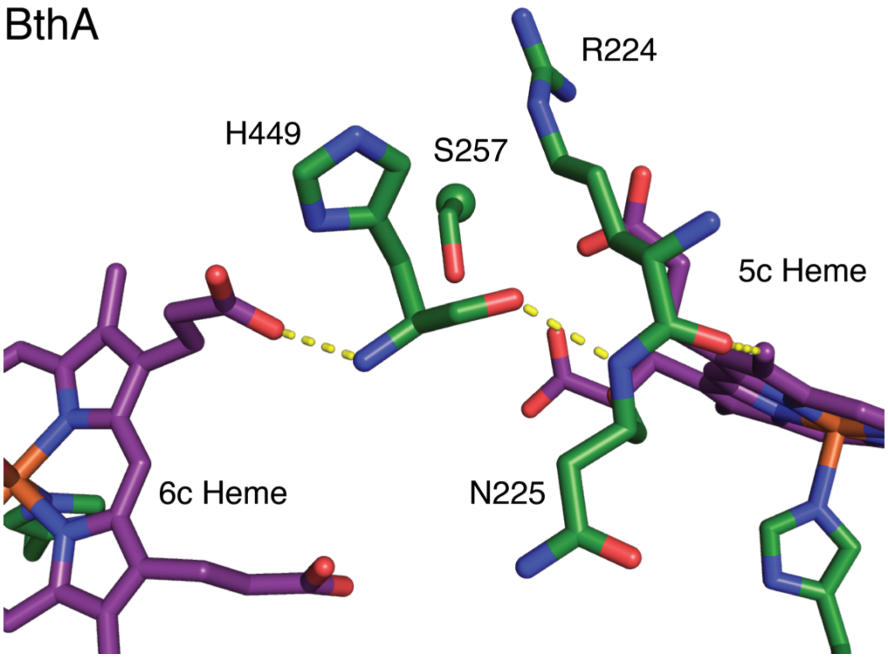

Supplementary Figure 8 | Alternate electron transfer residues involved in ET between 6c and 5c hemes as predicted by HARLEM. Interheme electron transfer pathway predicted by HARLEM/PATHWAYS for BthA, assuming a single step of ET from the 6c heme to the 5c heme. Through-space electron jumps denoted by yellow dashes.
